# *hsdS_A_* regulated extracellular vesicle-associated PLY to protect *Streptococcus pneumoniae* from macrophage killing via LAPosomes

**DOI:** 10.1128/spectrum.00995-23

**Published:** 2023-11-29

**Authors:** Liping Wang, Mengyuan Liu, Yixin Qi, Jian Wang, Qixue Shi, Xiaolin Xie, Changlin Zhou, Lingman Ma

**Affiliations:** 1 College of Life Science and Technology, China Pharmaceutical University, Nanjing, Jiangsu, China; Institut National de Santé Publique du Québec, Quebec, Canada

**Keywords:** *hsdS_A_*, extracellular vesicles, PLY, *Streptococcus pneumoniae*, LAPosomes

## Abstract

*Streptococcus pneumoniae* is a notorious human opportunistic pathogen which undergoes a spontaneous and reversible phenotypic change in response to the host environment. We demonstrated that the regulatory gene *hsdS_A_* of DNA methylation in the type I restriction modification system altered colony transparency and substantially contributed to *S. pneumoniae* virulence. Most importantly, *hsdS_A_* regulated the production of extracellular vesicles (EVs) which package cytosolic, surface, and secreted proteins, including pneumolysin (PLY). Interestingly, we confirmed that EV-associated PLY utilized internalization into macrophages to prolong the survival of intracellular bacteria as a major immune evasion strategy; that is, EV-associated PLY produced by the D39 strain (EVs-D39) could induce the formation of LC3-associated monolayer vacuoles [LC3-associated phagocytosis (LAP)] and co-localize with the NADPH oxidase 2 (NOX2) complex but not ULK1 when macrophages were infected with the D39Δ*ply* strain. In addition, EV-associated PLY derived from the EVs-D39 promoted macrophages to release more reactive oxygen species (ROS) and expression of p-p70s6k than EV-associated PLY derived from the D39Δ*hsdS_A_* strain (EVs-D39Δ*hsdS_A_*), whereas the expression of p-ULK1 was reversed, indicating that EVs-D39Δ*hsdS_A_* was more likely to induce conventional xenophagy. Furthermore, we identified the β1 integrin receptor as a crucial inducer of ROS to mediate LAP activation. Bacterial evasion of host clearance is closely related to insufficient acidification after the fusion of autophagosomes or LAPosomes with lysosomes. Of note, we found EV-associated PLY damaged the integrity of the lysosome membrane and changed the pH gradient, resulting in lysosomes being unable to remove intracellular bacteria and ultimately prolonging the survival of *S. pneumoniae* in macrophages. Finally, the extracted mouse alveolar macrophages and mouse intranasal infection models were employed to further verify the above findings.

**IMPORTANCE:**

*S. pneumoniae* is a major human pathogen that undergoes a spontaneous and reversible phase variation that allows it to survive in different host environments. Interestingly, we found *hsdS_A_*, a gene that manipulated the phase variation, promoted the survival and replication of *S. pneumoniae* in macrophages by regulating EV production and EV-associated PLY. More importantly, here we provided the first evidence that higher EV-associated PLY (produced by D39) could form LAPosomes that were single membrane compartments containing *S. pneumoniae*, which are induced by integrin β1/NOX2/ROS pathway. At the same time, EV-associated PLY increased the permeability of lysosome membrane and induced an insufficient acidification to escape the host killing, and ultimately prolonged the survival of *S. pneumoniae* in macrophages. In contrast, lower EV-associated PLY (produced by D39Δ*hsdS_A_*) activated ULK1 recruitment to form double-layered autophagosomes to eliminate bacteria.

## INTRODUCTION

*Streptococcus pneumoniae* is a notorious human opportunistic pathogen that is considered a serious threat to human health (1). Antibiotics and pneumococcal conjugate vaccines are the main means of clinical control of *S. pneumoniae* infectious diseases (2). However, the rise of drug-resistant bacteria caused by the abuse of antibiotics, prevalence of non-vaccine serotypes, and release of metabolites [such as membrane vesicles (MVs)] have greatly increased the difficulty of clinical treatment of pneumococcal diseases (3 – 5). Moreover, researchers showed that *S. pneumoniae* could also form intracellular bacteria in host cells through special mechanisms to evade immune surveillance and antibiotic killing (6).

As *S. pneumoniae* grows, it undergoes a spontaneous and reversible phenotypic change known as a phase variation. The change of colony transparency phenotype is the most typical characteristics of phase variation, which ultimately manifests as differences in bacterial virulence. Previous studies have shown that *hsdS_A_*, the dominant gene of the type I restriction modification system, mediates DNA methylation and alters the spontaneous colony transparency during *S. pneumoniae* invading host (7, 8). Its general appearance is that opaque colonies are more virulent with a thicker capsule and are more resistant to opsonophagocyte killing, whereas transparent colonies have thinner capsules and better adherence to host epithelial cells (9). Bacterial extracellular vesicles (EVs) are nanoscale (20–500 nm) lipid bilayer vesicles that influence host-pathogen interactions and are closely related to pathogen invasion and immune escape (10, 11). EVs of Gram-positive bacteria are mainly secreted by local plasma membrane expansion of living bacteria (12). Peptidoglycan (PGN) cross-linking and phenol-soluble modulins modulate EV production (13). *S. pneumoniae* EVs package diverse virulence proteins, such as pneumolysin (PLY), pneumococcal surface protein A (PspA) and pneumococcal adhesion and virulence protein B (14). Interestingly, we found that *hsdS_A_* was closely related to the contents of capsular polysaccharide (CPS) and PGN of *S. pneumoniae* by accident. For this, we hypothesized whether phase variation gene *hsdS_A_* would affect the release and components of EVs by changing the colony transparency phenotype.

In addition, more and more studies have shown that a variety of bacteria effectively secrete special proteins to inhibit the autophagy process of host cells, so as to avoid degradation of lysosomes and maintain intracellular survival and even proliferation (15, 16). The process of host removal of intracellular pathogens involves LC3-associated autophagy [LC3-associated phagocytosis (LAP)] and xenophagy. LAP, an atypical autophagy, is an LC3-associated phagocytic monolayer vacuolar induced by the assembly of NADPH oxidase NADPH oxidase 2 (NOX2) complexes and hypersecretion of reactive oxygen species (ROS) (17). In contrast, xenophagy is a bilayer structure autophagy induced by the ULK complex (18). LAP promotes the fusion of LAPosomes with acidic lysosomes to kill pathogens; importantly, it is also capable of transforming into classical autophagy (19). Interestingly, during the bactericidal process of LAP, some pore-forming toxins, such as listeriolysin O (20) and streptolysin O (18), target molecules involved in the formation of LAP through virulence factors, which are hidden in non-acidic LAPosomes to prevent recognition by x. Notably, Inomata et al. declared that *ply* gene knockout strains of *S. pneumoniae* have a reduced ability to induce the formation of LAP in mouse bone marrow-derived macrophages (BMDMs), but the detailed mechanism of action is still unclear (21).

In this study, we identified a phase variation gene, *hsdS_A_*, that played an important role in pathogen-host interactions of *S. pneumoniae*. The knockout of *hsdS_A_* decreased the production of EVs by upregulating the expression of PGN cross-linked genes, crucially reducing the PLY content in EVs. Moreover, we further demonstrated that EV-associated PLY induced NOX2 and LAP activation and reduced lysosome acidification to escape macrophage-mediated *S. pneumoniae* elimination. Our study provided critical insights into the role of *hsdS_A_* in EV production and characterized mechanisms by which EV-associated PLY prolonged the survival of *S. pneumoniae* in macrophages*,* which may provide novel targets and strategies for the treatment of *S. pneumoniae* infection.

## RESULTS

### Deletion of *hsdS_A_* converts pneumococcal phase variation and affects the contents of PGN and CPS

**Fig 1:**
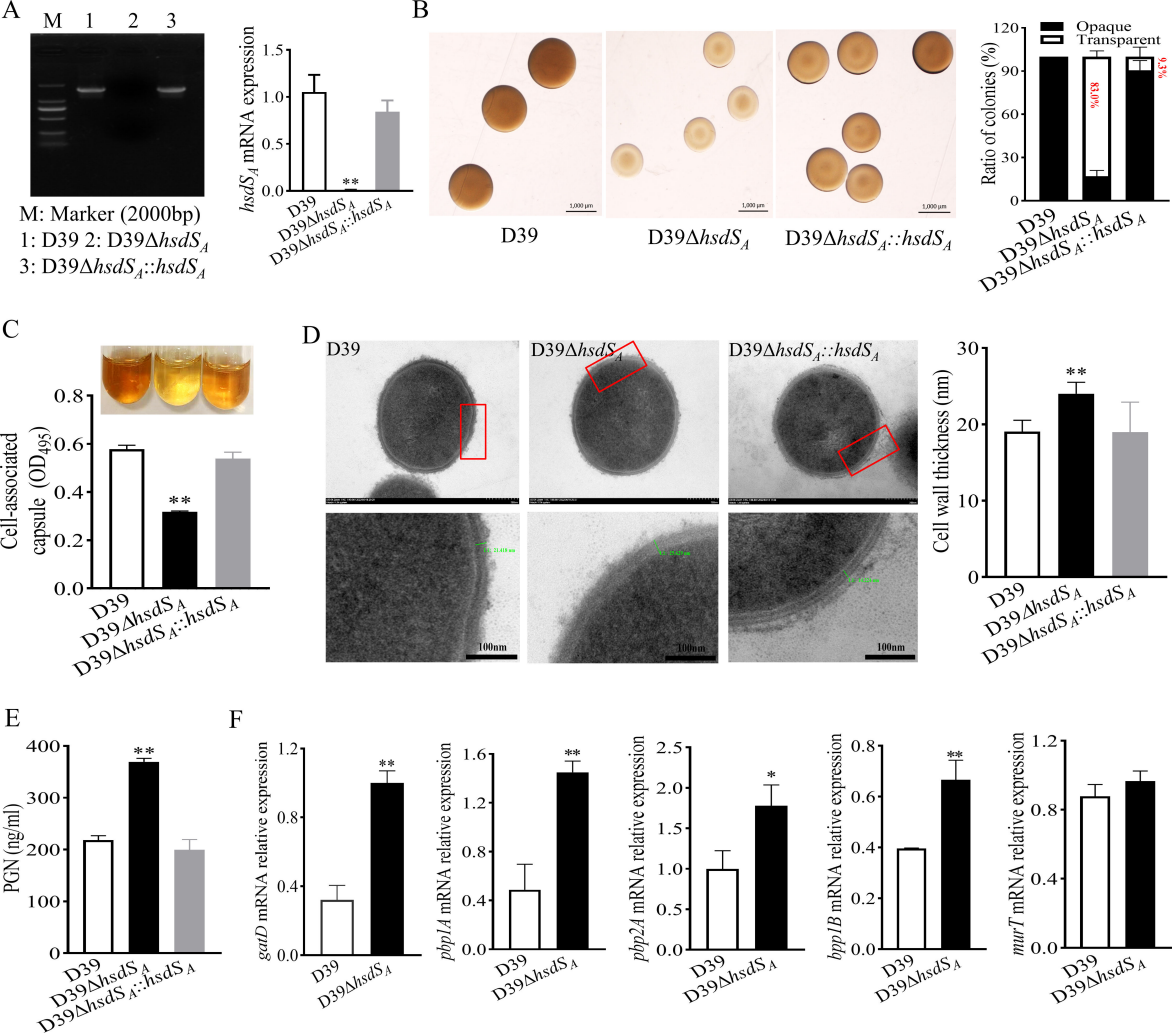
Effects of *hsdS_A_* knockout on the contents of CPS and PGN of *S. pneumoniae*. (**A**) Validation of *hsdS_A_* knockout efficiency by agarose gel electrophoresis and reverse transcription-quantitative real-time PCR (RT‒qPCR). Statistical analysis was analyzed by one-way analysis of variance (ANOVA) followed by Dunnett’s multiple comparison. *P<0.05, **P<0.01 vs D39. (**B**) Colony morphology of D39 and D39Δ*hsdS_A_* grown on TSA plates supplemented with catalase. The ratios of opaque and transparent colonies in each strain were compared. (**C**) Comparisons of whole bacterial CPS contents using uronic acid assay. Statistical analysis was analyzed by one-way ANOVA followed by Dunnett’s multiple comparison. *P<0.05, **P<0.01 vs D39. (**D**) Transmission electron microscopy results of representative D39 and D39Δ*hsdS_A_*. Mean cell wall thickness of 15 randomly chosen cells was measured by Image-Pro Plus. Statistical analysis was analyzed by one-way ANOVA followed by Dunnett’s multiple comparison. *P<0.05, **P<0.01 vs D39. (**E**) Comparisons of bacterial PGN using enzyme-linked immunosorbent assay. Statistical analysis was analyzed by one-way ANOVA followed by Dunnett’s multiple comparison. *P<0.05, **P<0.01 vs D39. (**F**) Relative mRNA expression levels of genes involved in peptidoglycan biosynthesis. Statistical analysis was analyzed by an unpaired Student *t*-test. *P<0.05, **P<0.01 vs D39.

Since the variation of *hsdS_A_* alleles switches pneumococcal phase variation, we engineered the *hsdS_A_* gene knockout strain through the introduction of the Janus cassette (22) into a streptomycin-resistant D39 derivative strain by homologous recombination (Fig. S1A). The resulting D39Δ*hsdS_A_* strain was validated by agarose nucleic acid gel electrophoresis and PCR (Fig. 1A). Subsequently, we detected that *hsdS_A_* knockout did not affect the growth of bacteria (Fig. S1B). To unequivocally determine the impact of *hsdS_A_* on the phase variation known as switch between transparent and opaque phenotypes of pneumonia, we observed the colony morphology on soybean casein digest agar (TSA) plates supplemented with catalase under a dissection microscope. Microscopic examination revealed that the parental strain D39 (wild-type D39 with streptomycin-resistance, D39) was uniformly opaque colonies (100%), after 24 h of culture. In sharp contrast, the *hsdS_A_* deletion strain yielded large numbers of transparent colonies (83%) with occasional formation of opaque colonies (17%). However, the D39Δ*hsdS_A_::hsdS_A_* strain, an *hsdS_A_* complement strain, was composed of 90.7% opaque and 9.3% transparent colonies (Fig. 1B), indicating a strong correlation between *hsdS_A_* and colony transparency.

Previous reports have indicated that the opaque phenotype had greater amounts of capsule contributing to more virulence, while the transparent phenotype had more immunodetectable teichoic acid contributing to asymptomatic colonization (7, 23). To verify whether *hsdS_A_* knockout affected the CPS and PGN contents of *S. pneumoniae*, we further detected CPS contents via uronic acids. Indeed, the D39 and complemental strain exhibited a significantly elevated OD_495_ (0.58 ± 0.07, 0.54 ± 0.02) representing the polysaccharide content, compared to *hsdS_A_* knockout strain (0.32 ± 0.013) (Fig. 1C). To further confirm the effects of *hsdS_A_* on the PGN and cell wall, the bacterial cell wall thickness of 15 randomly chosen cells were observed by transmission electron microscopy (TEM) and Image-Pro Plus, and the content of PGN was determined *via* sandwich enzyme-linked immunosorbent assay (ELISA), respectively. As shown in Fig. 1D, *hsdS_A_* knockout led to a significantly increased cell wall thickness (24.0 ± 0.95 nm) compared with D39 (19.05 ± 0.97 nm) and D39Δ*hsdS_A_::hsdS_A_* (18.99 ± 1.09 nm). Interestingly, a similar comparison of the amount of PGN displayed that the *hsdS_A_* knockout strain produced up to 368.64-ng/mL PGN in an equivalent bacterial suspension, which was higher than that of D39 (216.02 ng/mL) and the complemental strain (199.77 ng/mL) (Fig. 1E). Additionally, one potential mechanism for the elevated PGN production may be attributed to the expression of biosynthesis-related genes, including *pbp1A*, *pbp1B*, *pbp2A*, *gatD*, and *murT*. As expected, *hsdS_A_* deletion resulted in an obvious increase in the expression of *pbp1A*, *pbp1B*, *pbp2A*, and *gatD* (Fig. 1F). The data above suggested that *hsdS_A_* knockout could promote the phase variation toward transparent colonies, reduce CPS content, and enhance PGN production and pneumococcal cell wall thickness.

### The *hsdS_A_* gene is important for pneumococcal infection and virulence

**Fig 2:**
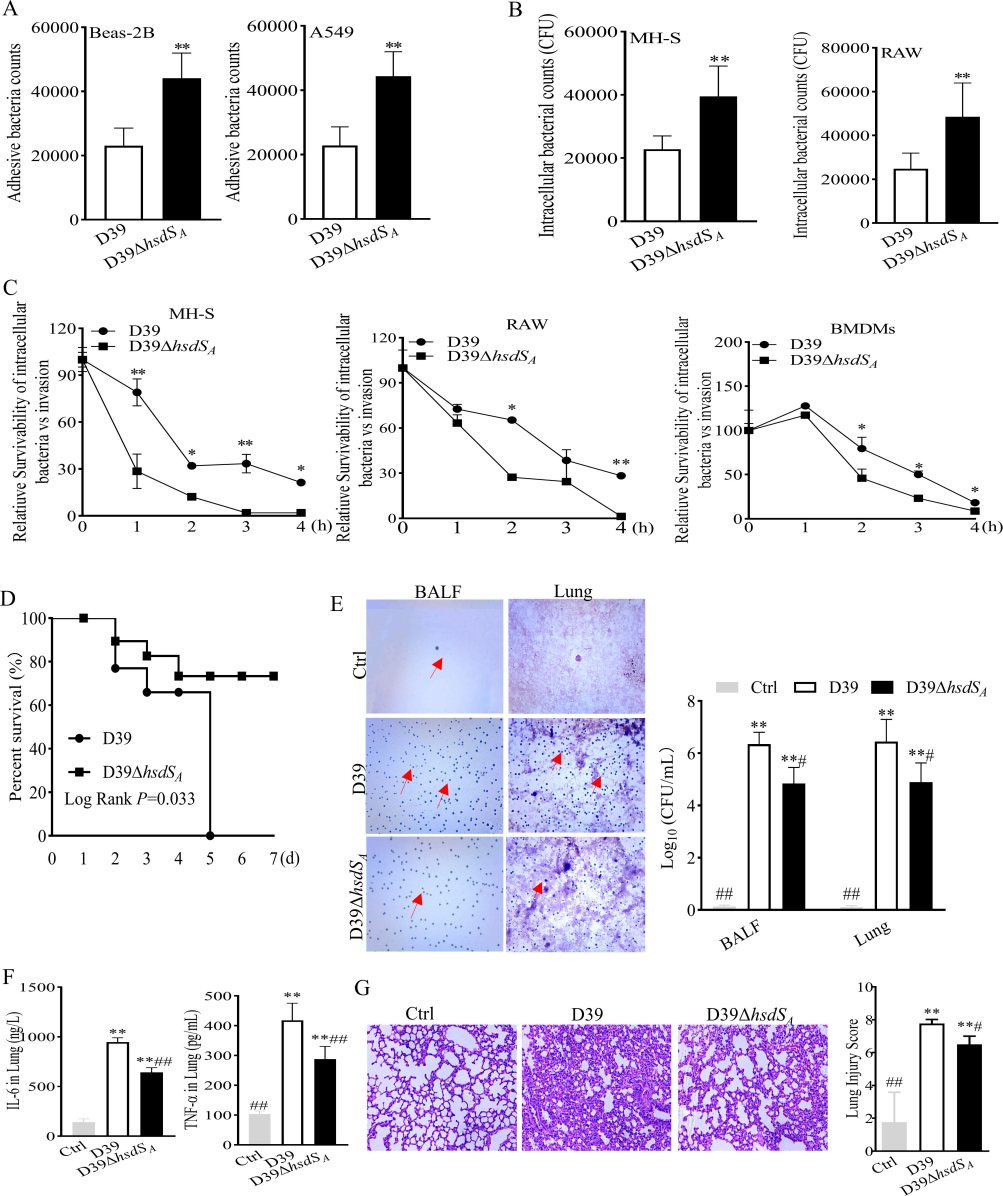
The *hsdS_A_* gene is important for pneumococcal infection and virulence. After infecting cells with an MOI of 20 *S*. *pneumoniae*, (**A**) the adhesion ability of D39 and D39Δ*hsdS_A_* toward epithelial cells (Beas-2B and A549 cell lines) and (**B**) their anti-phagocytic ability against macrophages (MH-S and RAW264.7 cell lines) were evaluated by plate count methods. For anti-phagocytic ability detection, extracellular bacteria were killed by antibiotics. Statistical analysis was analyzed by an unpaired Student *t*-test. *P<0.05, **P<0.01 vs D39. (**C**) The intracellular survival of bacteria at the indicated time points after extracellular bacteria killing by antibiotics and cells undergoing continuous culture. Statistical analysis was analyzed by an unpaired Student *t*-test. *P<0.05, **P<0.01 vs D39. (**D**) Percent survival experiments implemented in mice intranasally inoculated with D39 or D39Δ*hsdS_A_* (1 × 10^8^ CFU). Statistical analysis was analyzed by Kaplan-Meier. *P<0.05, **P<0.01 vs D39. (**E**) Plate count methods and Gram staining to determine the bacterial loads in bronchoalveolar lavage fluid (BALF) and mouse lung homogenate; red arrows are labeled *S. pneumoniae*. Statistical analysis was analyzed by one-way ANOVA followed by Dunnett’s multiple comparison. *P<0.05, **P<0.01 vs Ctrl; ^#^ *P* < 0.05, ^##^ *P* < 0.01 vs D39. (**F**) ELISA for the contents of interleukin-6 (IL-6) and tumor necrosis factor alpha (TNF-α) in mouse lung tissue homogenate. Statistical analysis was analyzed by one-way ANOVA followed by Dunnett’s multiple comparison. *P<0.05, **P<0.01 vs Ctrl; ^#^ *P* < 0.05, ^##^ *P* < 0.01 vs D39. (**G**) Hematoxylin and eosin staining for mouse lung histopathological examination after *S. pneumoniae* infection. (Right panel) The scores of lung tissue injury analyzed by a professional pathologist. Statistical analysis was analyzed by one-way ANOVA followed by Dunnett’s multiple comparison. *P<0.05, **P<0.01 vs Ctrl; ^#^ *P* < 0.05, ^##^ *P* < 0.01 vs D39.

Opaque variants of *Streptococcus* are more virulent in animal models but colonize the nasopharynx poorly, while transparent variants colonize the nasopharynx more efficiently but are relatively avirulent (9, 24, 25). To determine the role of *hsdS_A_* during infection, MH-S and RAW264.7 cells were infected with D39 and D39Δ*hsdS_A_* strains, respectively, at a high multiplicity of infection (MOI) (40:1). The morphology of D39-infected cells showed obvious shrinkage and rupture in contrast to the nearly intact cells infected by D39Δ*hsdS_A_* after 3 h of coincubation (Fig. S2A). Moreover, the cell death rate of D39 strain-infected cells at MOI = 40 was higher than that of control and MOI = 20 (Fig. S2B). Thus, the adhesion and anti-phagocytosis effects of D39 and D39Δ*hsdS_A_* toward epithelial cells and macrophages were evaluated at a relatively low MOI (20:1). As shown in Fig. 2A and B, D39Δ*hsdS_A_* showed increased counts of bacteria attaching to epithelial cells and invading into macrophages after 2 h of infection. Moreover, intracellular survivability assays were performed in MH-S and RAW264.7 cells to evaluate whether *hsdS_A_* impacts bacterial replication in macrophages. The results showed that the survival of D39Δ*hsdS_A_* strain was lower than that of D39 strain at the stage of continued infection of an intracellular bacterium within host cells. This phenomenon was further confirmed in BMDMs (Fig. 2C). These data indicated that the *hsdS_A_* gene was conducive to the replication of *S. pneumoniae* D39 in macrophages.

Furthermore, the survival rate of mice infected intranasally with 1 × 10^8^ CFU D39 strain showed a rapid drop until death after day 6. In contrast, mice infected intranasally with D39Δ*hsdS_A_* maintained a survival rate of 73.4% until the end of the experiment, which was higher than that of the D39 infection group (Fig. 2D). Subsequently, during the invasive period, the counts of bacteria in bronchoalveolar lavage fluid (BALF) and lung tissue from D39-infected mice were higher than those from D39Δ*hsdS_A_*-infected mice at 24 h after infection (Fig. 2E). In addition, since interleukin (IL)-6 and tumor necrosis factor alpha (TNF-α) are considered to be important contributors to the development of lung injury due to their potent inflammatory activity (26), we also measured their contents in mice lung homogenates and found that the concentrations of IL-6 and TNF-α in the D39-infected group were much higher than those of D39Δ*hsdS_A_*-infected group (Fig. 2F). Consequently, the former mouse presented more severe lung lesions and higher lung injury scores, including pulmonary edema, congestion, and inflammatory cell infiltration (Fig. 2G). These observations demonstrate that the opaque colony derived from D39 had a stronger invasion and virulence due to *hsdS_A_* gene regulation.

### Isolation and protein composition of *S. pneumoniae* EVs

**Fig 3:**
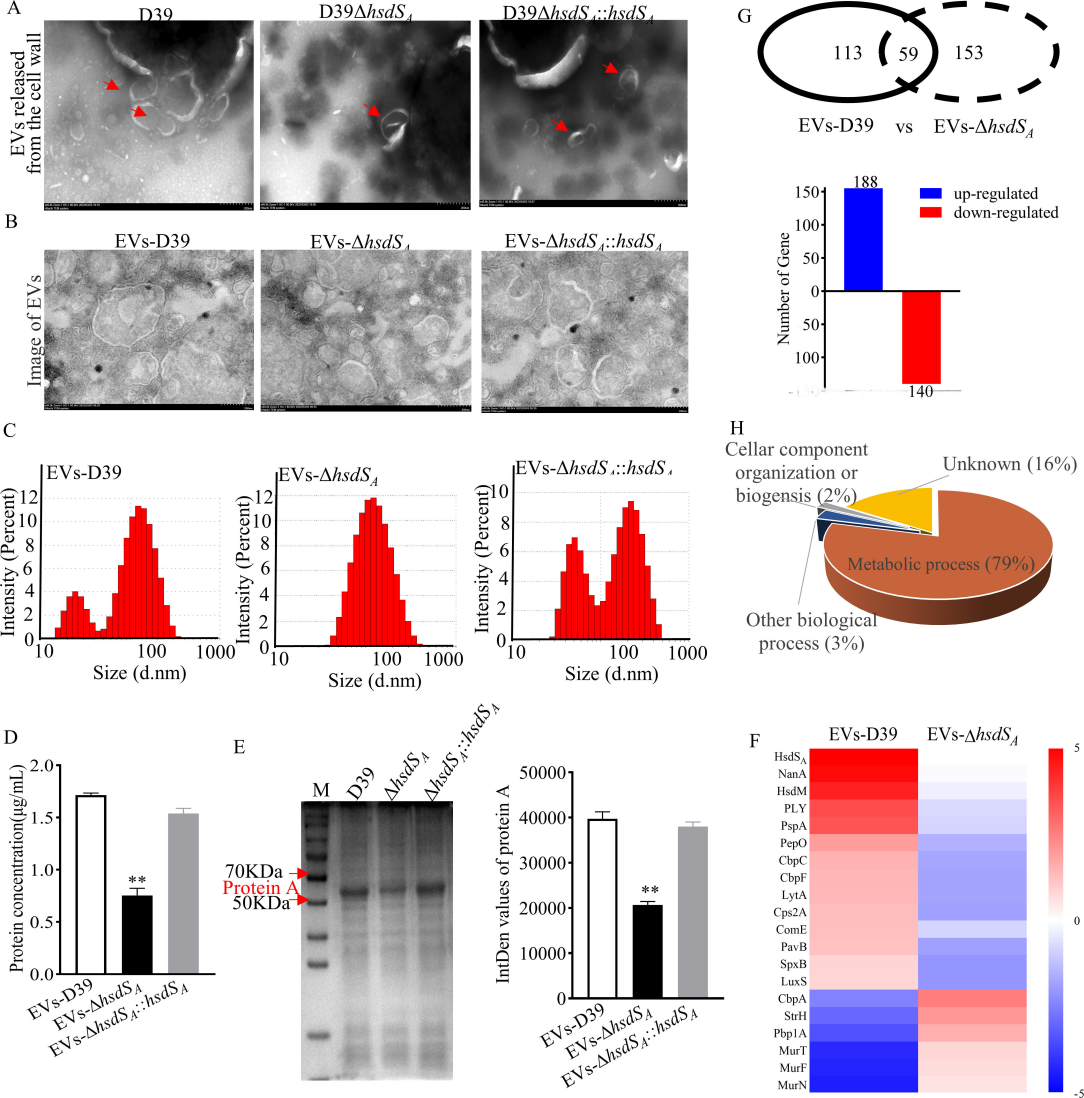
Isolation and protein composition of *S. pneumoniae* EVs. TEM assay of (**A**) bacterial suspensions from D39, D39Δ*hsdS_A_*, and D39Δ*hsdS_A_::hsdS_A_* at logarithmic phase to observe the budding of vesicle-like structures from the *S. pneumoniae* cell surface (red arrows indicate EVs) and (**B**) isolated EVs from the culture supernatant of these three strains by ultracentrifugation. (**C**) Nanobrook ZetaPALS potential analyzer for detecting the particle size. The total protein concentrations and ingredient fractions contained in EVs were detected by BCA (bicinchoninic acid) protein assay kit (**D**) and SDS-PAGE. Statistical analysis was analyzed by one-way ANOVA followed by Dunnett’s multiple comparison. **P<0.01 vs EVs-D39. (**E**) Quantitative proteomic analysis of the protein composition difference in EVs after *hsdS_A_* knockout. (**F**) Heatmap depicting the selected virulence factors enriched in EVs. (**G**) Statistics of protein contents and the number of genes upregulated or downregulated in EVs and (**H**) statistics of protein functions in EVs.

EVs are nanosized, spherical, naturally occurring lipid bilayer vesicles with a cargo that includes diverse proteins, nucleic acids, and PGN known as microbe-associated molecular patterns (12, 27). Previous studies have shown that PGN cross-linking and autolysin activity modulate EV production by altering the permeability of cell walls (28). Here, we confirmed that *hsdS_A_* knockout increased the content of PGN and upregulated the expression of genes involved in biosynthesis of PGN. This raised an interesting hypothesis regarding whether *hsdS_A_* knockout also affected EV production and the question of whether the changed EVs would interfere with pathogen-host interactions. Firstly, EVs were isolated by concentrating the culture supernatants harvested from logarithmic to remove small molecules (<100 kDa) before ultracentrifugation. These isolated EVs were visualized by TEM, and particle size was detected by a nanoparticle size analyzer. The morphology showed that EVs were a bilayer membrane structure of nanoparticles (Fig. 3A). EVs-D39 showed two peaks with an average particle size of 110 nm. Comparatively, EVs-D39Δ*hsdS_A_* had only one peak with an average particle size of 85 nm (Fig. 3B; Fig. S2C). Further protein concentration and ingredient fractions subjected to SDS-PAGE displayed that EVs-D39 contained more protein than EVs-D39Δ*hsdS_A_* under the same amounts of bacteria. Attentively, there was a markedly differential protein A between 50 and 70 kDa (Fig. 3C and D).

Finally, in order to better investigate the protein composition difference of EVs resulting from *hsdS_A_* knockout, quantitative proteomic analysis was performed. As shown in Fig. 3E through G, *hsdS_A_* knockout resulted in 328 differentially expressed proteins in EVs that are mainly associated with metabolic processes (79%), among which 188 proteins were upregulated and 140 proteins were downregulated. Moreover, EVs contained many proteins associated with virulence, sharing the significant differential proteins caused by *hsdS_A_* knockout on a heatmap while also having unique proteins, including PBP1A, MurT, MurF, and PBP1B related to the synthesis and crosslinking of PGN. Given the importance of virulence in *S. pneumoniae* pathogenesis, such as PLY (29), we further explored vesicle-associated virulence factor composition with a heatmap for different strains. Hereinto, neuraminidase (NanA), PLY, and PspA were three virulence factors with the most obvious difference in EVs.^30^); PLY is a controversial virulence factor in regulating pathogen-host interactions, because some researchers believe that PLY may help bacteria escape from host immune cell elimination, while others just argue the opposite view (21, 31). PspA interferes with complement-mediated regulatory phagocytosis by reducing the deposition of C3b on the *S. pneumoniae* surface (30). In our study, we mainly focused on the role of EV-associated PLY in intracellular bacterial elimination.

### Cytotoxicity and internalization assays of EV-associated PLY in macrophages

**Fig 4:**
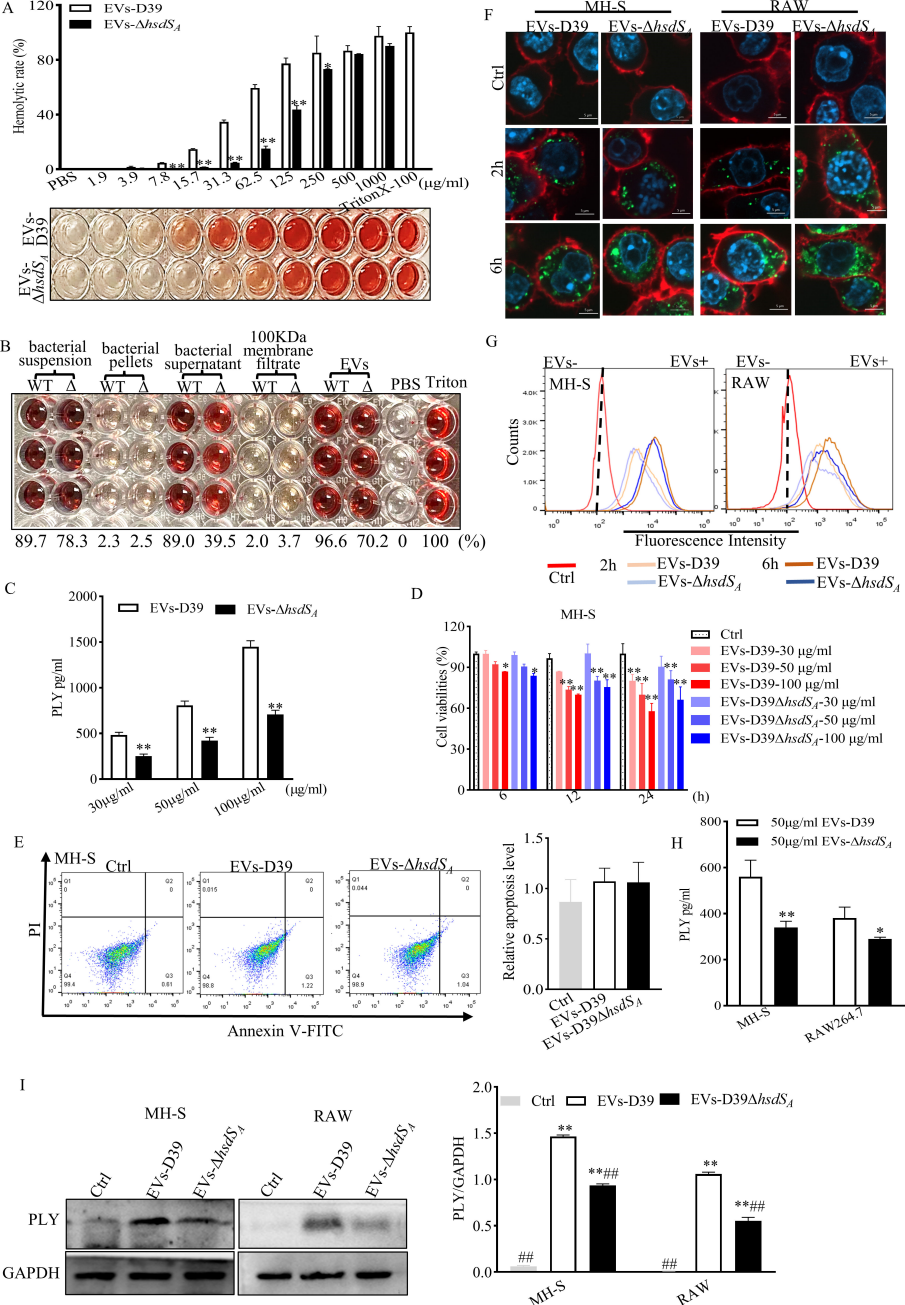
Cytotoxicity and internalization assays of EV-associated PLY in macrophages. (**A**) Observation of 2% RBC hemolysis with different concentrations of EVs. Hemolysis rates were calculated by the absorbance at 450 nm. Statistical analysis was analyzed by an unpaired Student *t*-test. *P<0.05, **^*^ *P*<0.01 vs EVs-D39. (**B**) The hemolysis activity of the whole bacterial suspension, bacterial pellets, bacterial supernatant, and 100-kDa membrane filtrate produced in the EVs-D39 extraction process. (**C**) ELISA detection of PLY contents derived from EVs. Statistical analysis was analyzed by an unpaired Student *t*-test. *P<0.05, **P<0.01 vs EVs-D39. (**D**) MTT assay for macrophage cell viability in the presence of different concentrations of EVs for 6, 12, and 24 h of coincubation. Statistical analysis was analyzed by one-way ANOVA followed by Dunnett’s multiple comparison. *P<0.05, **P<0.01 vs Ctrl. (**E**) Representative flow cytometry analysis and quantification of macrophage apoptosis with 100-µg EV stimulation for 24 h. Statistical analysis was analyzed by one-way ANOVA followed by Dunnett’s multiple comparison. *P<0.05, **P<0.01 vs Ctrl. (**F**) Representative confocal images showing the internalization of EVs (100 µg) into macrophages at different time points. Green, DiO-stained EVs; red, cytomembrane; blue, 2-(4-amidinophenyl)-6-indolecarbamidine dihydrochloride-stained nuclei. (**G**) Flow cytometry measurements of the internalization efficiency of macrophages toward EVs (100 µg) at different time points. ELISA (**H**) and Western blot assays for PLY contents in macrophages derived from the internalization of EVs (100 µg) for 6 h (**I**). Statistical analysis was analyzed by an unpaired Student *t*-test or one-way ANOVA followed by Dunnett’s multiple comparison. *P<0.05, **P<0.01 vs EVs-D39 or *P<0.05, **P<0.01 vs Ctrl; ^##^ *P* < 0.01 vs EVs-D39. FITC, fluorescein isothiocyanate; WT, wild type.

Because it lacks a typical N-terminal signal for secretion, PLY was previously considered to be a virulent factor released during bacterial autolysis. However, in recent years, it has also been reported that PLY can be located in the *S. pneumoniae* cell wall and is utilized by extracellular proteases or enriched in EVs (14, 32). In this study, we collected the components (whole bacterial suspension, bacterial pellets, bacterial supernatant, and 100-kDa membrane filtrate) produced in the EVs-D39 extraction process and incubated them with 2% red blood cell (RBC) to clarify where PLY was present. Surprisingly, whole bacterial suspension, bacterial supernatant, and EVs exhibited obvious hemolytic activity compared to that of phosphate buffered saline (PBS) (Fig. 4B), suggesting that PLY ultimately exists in EVs. The results are consistent with previous studies (33, 34). Additionally, we further designed a series of hemolysis experiments with different concentrations of EVs and observed that the hemolytic activity of EVs was increased in a dose-dependent manner (Fig. 4A). Notably, the hemolytic activity of EVs-D39 was approximately threefold higher than that of EVs-D39Δ*hsdS_A_* at the same EVs concentration. Moreover, the contribution of PLY in the same concentration of EVs was also examined. As shown in Fig. 4C, PLY contents of 30-, 50-, and 100-µg/mL EVs-D39 were found to be 1.9-, 1.9-, and 2.0-folds higher than those of EVs-D39Δ*hsdS_A_*, respectively. Likewise, MH-S and RAW cells incubated with EVs-D39Δ*hsdS_A_* for 24 h had higher cell viabilities. In contrast, the EVs at 30 or 50 µg/mL had no effect on cell viability after 6 h of coincubation (Fig. 4D; Fig. S3A). These data suggest that *hsdS_A_* knockout resulted in a reduced PLY content associated with *S. pneumoniae* EVs, which further gave rise to reduction of cytotoxicity. However, cells incubated with 50-µg/mL EVs for 24 h did not show significant apoptosis (Fig. 4E; Fig. S3B).

Although numerous studies have reported that *S. pneumoniae* EVs can be internalized by lung epithelial cells, monocyte-derived dendritic cells, and mouse macrophages (J774A.1) (14, 30), whether *hsdS_A_* knockdown affects EV internalization into mouse macrophages remains unknown. To this end, EVs were labeled with 3,3′-dioctadecyloxacarbocyanine perchlorate (DiO), a green fluorescent lipophilic membrane dye, has been widely used to track the internalization of exosomes. Similarly, macrophage membranes were stained with 1,1′-dioctadecyl-3,3,3′,3′-tetramethylindodicarbocyanine,4-chlorobenzenesulfonate salt (DiD), which is a commonly used red cell membrane dye. DiO-labeled EVs (50 µg/mL) were exposed to MH-S and RAW for 2 or 6 h and were assessed by confocal microscopy and flow cytometry. As shown in Fig. 4F and G, EVs with fluorescence were internalized into macrophages in a time-dependent manner, but nonsense *hsdS_A_* knockout to it, the internalization of these two kinds of EVs were nearly the same (Fig. 4F and G), suggesting that the bacterial virulence protein constituents packaged by EVs are delivered into host cells. On account of this, it was speculated that PLY was also largely carried into host cells by EVs. To validate this conjecture, Western blotting and ELISA kits were used to examine PLY content in macrophages after being co-incubated with 50-µg/mL EVs for 6 h. Prior to analysis, the free EVs were washed away with pre-cooled PBS. Consistent with dada above, EV-associated PLY was indeed internalized into macrophages, and PLY derived from EVs-D39 (560.6 pg/mL in MH-S cells and 380.8 pg/mL in RAW cells) was higher than that derived from EVs-D39Δ*hsdS_A_* (340.6 pg/mL in MH-S cells and 289.4 pg/mL in RAW cells) (Fig. 4H). This phenomenon was further confirmed by Western blotting (Fig. 4I). Together, these data indicate that PLY of *S. pneumoniae* can be enriched in EVs and internalized into macrophages, and *hsdS_A_* knockout reduced the amount of PLY in EVs.

### EV-associated PLY induced NOX2 and LAP activation

**Fig 5:**
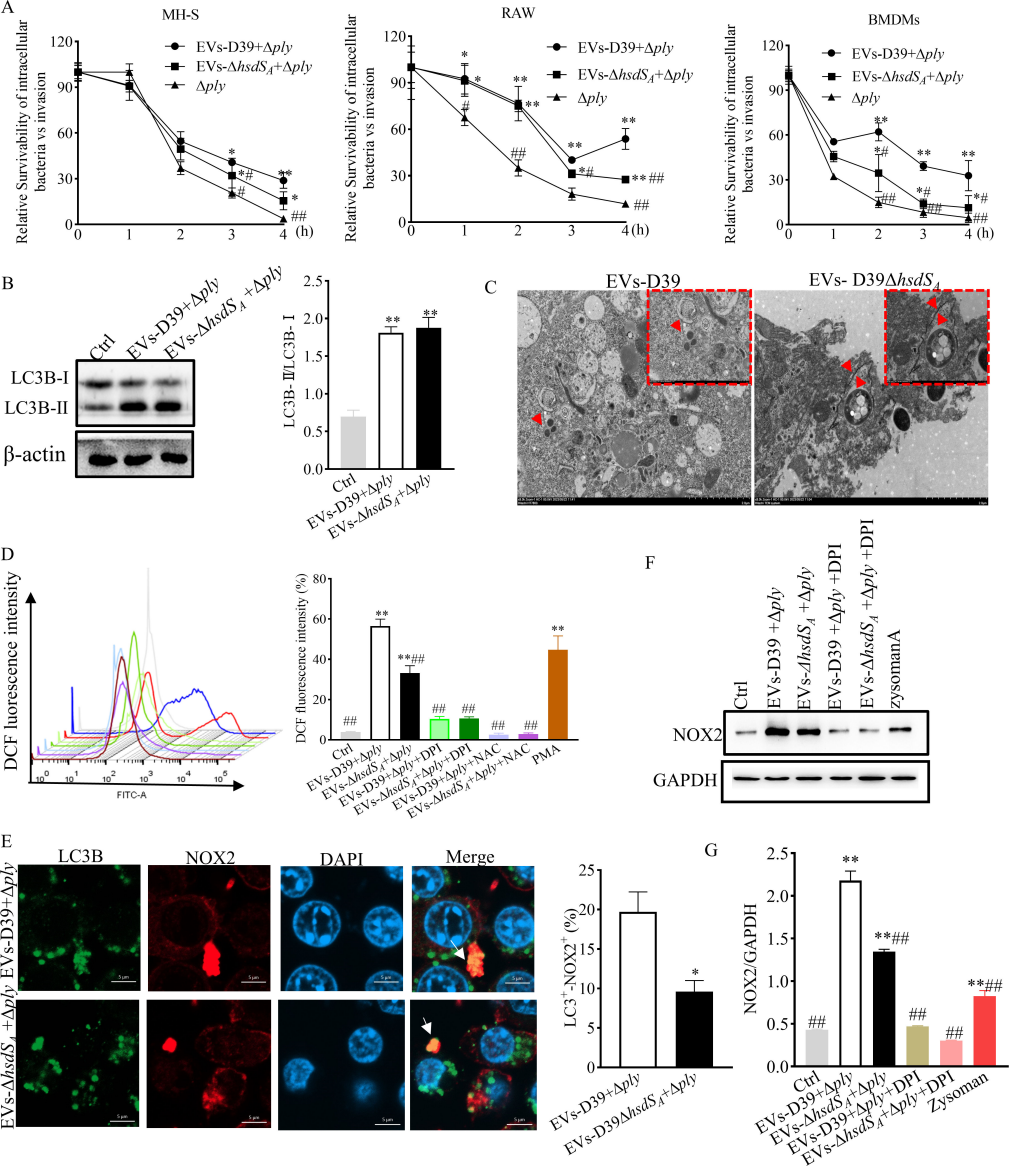
EV-associated PLY induced NOX2 and LAP activation. (**A**) The intracellular survival of D39Δ*ply* in macrophages pre-treated with D39-derived EVs or D39Δ*hsdS_A_*-derived EVs. Statistical analysis was analyzed by one-way ANOVA followed by Dunnett’s multiple comparison. *P<0.05, **P<0.01 vs D39Δ*ply*, ^#^ *P* < 0.05, ^##^ *P* < 0.01 vs EVs-D39 + D39Δ*ply*. (**B**) Western blot assay for the conversion of LC3-I to LC3-II in macrophages pre-treated with EVs for 4 h and infected with D39Δ*ply* for 2 h. Statistical analysis was analyzed by one-way ANOVA followed by Dunnett’s multiple comparison. *P<0.05, **P<0.01 vs Ctrl. (**C**) TEM results of the membrane structure of phagosomes (membrane surrounding D39Δ*ply* in macrophages) after bacterial infection. White arrow shows the phagosome membrane. Red indicates the autophagosome membrane. Macrophages were pre-treated with EVs for 4 h and then infected with D39Δ*ply* for 2 h. (**D**) Representative flow cytometry analysis of ROS release (right panel, quantification of DCF [2',7'-dichlorofluorescein] fluorescence intensity). Statistical analysis was analyzed by one-way ANOVA followed by Dunnett’s multiple comparison. *P<0.05, **P<0.01 vs Ctrl; ^#^ *P* < 0.05, ^##^ *P* < 0.01 vs EVs-D39 + D39Δ*ply*. (**E**) Immunofluorescence assay for the co-localization of LC3B-I/II and NOX2 and quantification was performed by Image J analysis; White arrows represent LC3 co-localization points with NOX2. Statistical analysis was analyzed by an unpaired Student *t*-test. *P<0.05, **P<0.01 vs EVs-D39 + D39Δ*ply*. (**F and G**) Western blot assay for the expression levels of NOX2. Statistical analysis was analyzed by one-way ANOVA followed by Dunnett’s multiple comparison. *P<0.05, **P<0.01 vs Ctrl; ^#^ *P* < 0.05, ^##^ *P* < 0.01 vs EVs-D39 + D39Δ*ply*.

Recombinant PLY inhibits the maturation of autophagosomes in human brain microvascular endothelial cells, resulting in *S. pneumoniae* R6 or TIGR4 in vacuoles to maintain intracellular survival (29). Our results showed that D39 was taken up by macrophages and could survive significantly longer, even replicate in BMDMs (Fig. 2C). To clarify whether EV-associated PLY could mediate intracellular survival of D39 and how it acted, macrophages were respectively pre-treated with 50-µg/mL EVs-D39 or EVs-D39Δ*hsdS_A_* for 4 h, and the intracellular survival of D39Δ*ply* was statistically counted at different time points. As expected, when compared with the no-EVs-added group, the pre-treatment with these two kinds of EVs both enhanced the survival ability of D39Δ*ply*, particularly at later time points of 3 and 4 h. Importantly, under EVs-D39 pre-treatment, D39Δ*ply* showed 2.0-, 1.4-, and 3.0-folds higher survival efficiency than EVs-D39Δ*hsdS_A_* pre-treated groups in MH-S, RAW, and BMDMs, respectively (Fig. 5A), which may be mainly attributed to the higher enrichment of PLY in EVs-D39. This suggests a particular role of EV-associated PLY in intracellular survival of *S. pneumoniae* during macrophage infection.

**Fig 6:**
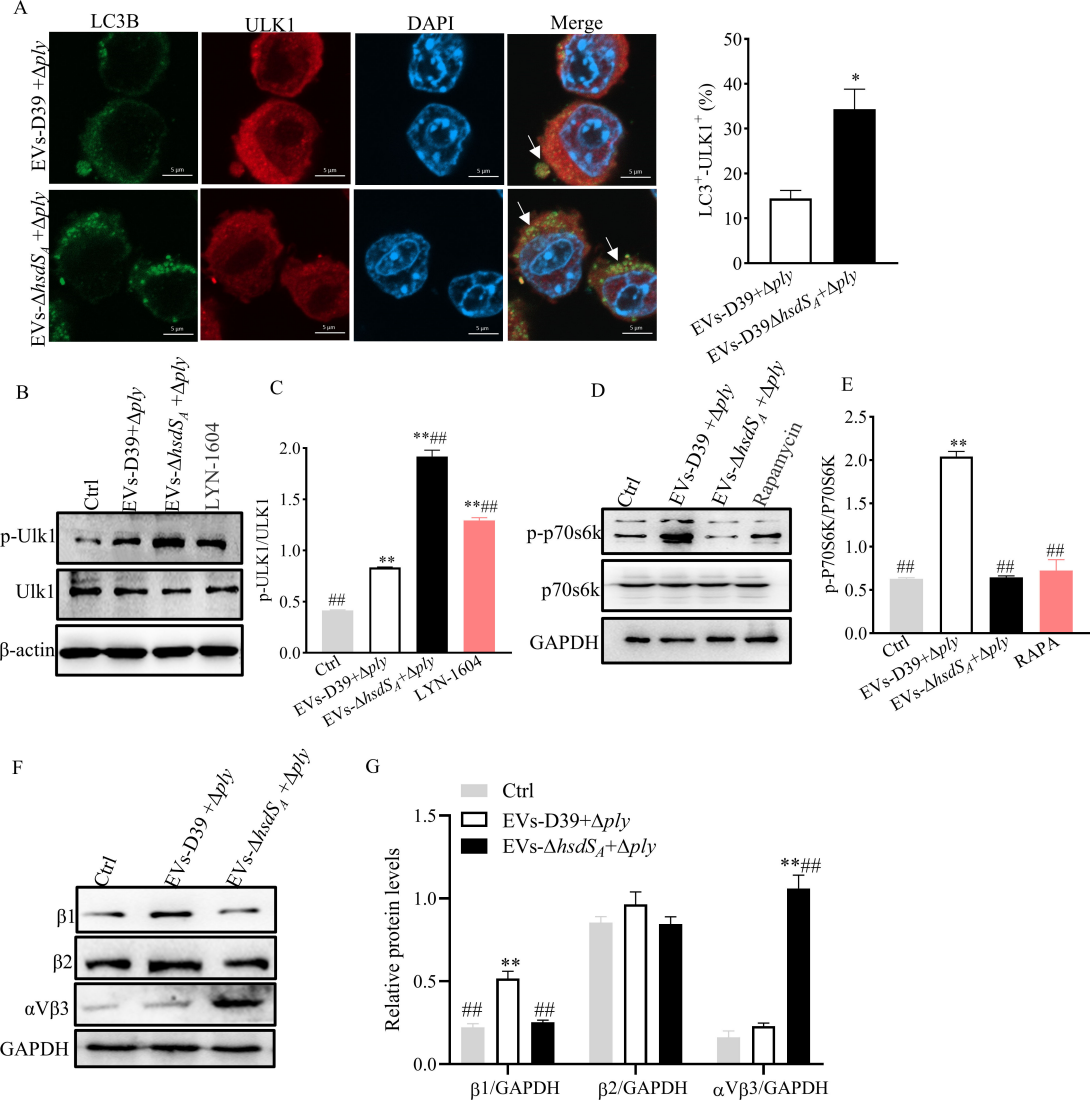
Effect of EV-associated PLY on xenophagy formation. (**A**) Immunofluorescence assay for the co-localization of LC3B-I/II and ULK1 and quantification was performed by Image J analysis; white arrows represent LC3 co-localization points with ULK1. Statistical analysis was analyzed by an unpaired Student *t*-test. *P<0.05, **P<0.01 vs EVs-D39 + D39Δ*ply*. (**B–G**) Western blot assay for the expression levels of p-ULK1, p-P70S6K, and integrins β1, β2, and αVβ3. Statistical analysis was analyzed by one-way ANOVA followed by Dunnett’s multiple comparison. *P<0.05, **P<0.01 vs Ctrl; ^##^ *P* < 0.01 vs EVs-D39 + D39Δ*ply*.

Next, conversion of the cytosolic form of LC3 (LC3-I) to the membrane-associated form of LC3 (LC3-II) was indistinguishable under these two kinds of EVs pre-treatment in both MH-S and RAW cells (Fig. 5B; Fig. S5A). LAP and xenophagy are involved in the process of host removing intracellular debris. A hallmark of LAP is the recruitment of LC3 to the single membrane phagosomes, which requires ROS production by the NADPH oxidase NOX2 (35). In contrast, xenophagy is activated by ULK complex components to form a bilayer membrane autophagosome. To investigate which of these two pathways was activated by EV-associated PLY, we analyzed the membrane structure surrounding D39Δ*ply* in macrophages after 4 h of EV pre-treatment. As shown in Fig. 5C, the majority of the monolayer structure surrounding D39Δ*ply* appeared in EVs-D39 pre-treated macrophage; however, a large amount of double-layered structure surrounding D39Δ*ply* appeared in EVs-D39Δ*hsdS_A_* pre-treated macrophages. Subsequently, the co-localization of NOX2 and LC3, as well as ROS production, was detected after 4 h of EV pre-treatment and 2 h of D39Δ*ply* infection. The flow analysis results showed that ROS production in the EVs-D39 pre-treated group (56.56% versus 78.2%) was significantly higher than that in the EVs-D39Δ*hsdS_A_* pre-treated group (33.20% versus 59.05%) in MH-S and RAW264.7 cells, respectively (Fig. 5D; Fig. S5B). However, after treatment with NOX2-selective inhibitor diphenyleneiodonium (DPI) or ROS scavenger *N*-acetyl-L-cysteine, ROS production was markedly reduced and the difference caused by these two kinds of EVs disappeared (Fig. 5D). Similarly, results from immunofluorescence and Western blotting also confirmed that NOX2 puncta were significantly concentrated on LC3 (Fig. 5E through G; Fig. S5C), and NOX2 expression was highest in the presence of EVs-D39 pre-treatment but without DPI intervention (Fig. 5F and G; Fig. S5D and E). Moreover, D39Δ*ply* failed to increase NOX2 expression without pre-treatment with EVs (Fig. S5F and G). To confirm whether EVs-D39Δ*hsdS_A_* pre-treatment could activate canonical autophagy, we determined the co-localization of ULK1 and LC3 and the expression of p-ULK1. Results showed that ULK1/LC3 co-localization and ULK1 phosphorylation were all significantly increased by the pre-treatment of EVs-D39Δ*hsdS_A_* but not EVs-D39 (Fig. 6A through C; Fig. S6A and B), indicating the activation of canonical autophagy. Initiation of canonical autophagy requires the inactivation of mTOR, which results in dephosphorylation of the mTOR substrate p70S6K (36). As shown in Fig. 6D and E, p-P70S6K remained at a low level. The data were verified equally on RAW cells (Fig. S6C). To search for the host cell receptor mediating EV-associated PLY-induced NOX2 and LAP activation, we next focused on β1, β2, and αVβ3 integrins which associated with LAP previously reported (18). Using Western blotting, we found that only β1 integrin, but not β2 integrin and αVβ3 integrin, was increased under EVs-D39 pre-treatment (Fig. 6F and G). This indicates that EV-associated PLY can induce NOX2 and LAP activation via the β1 integrin receptor.

### EV-associated PLY reduced lysosome acidification to evade host immune defenses for *S. pneumoniae*

**Fig 7:**
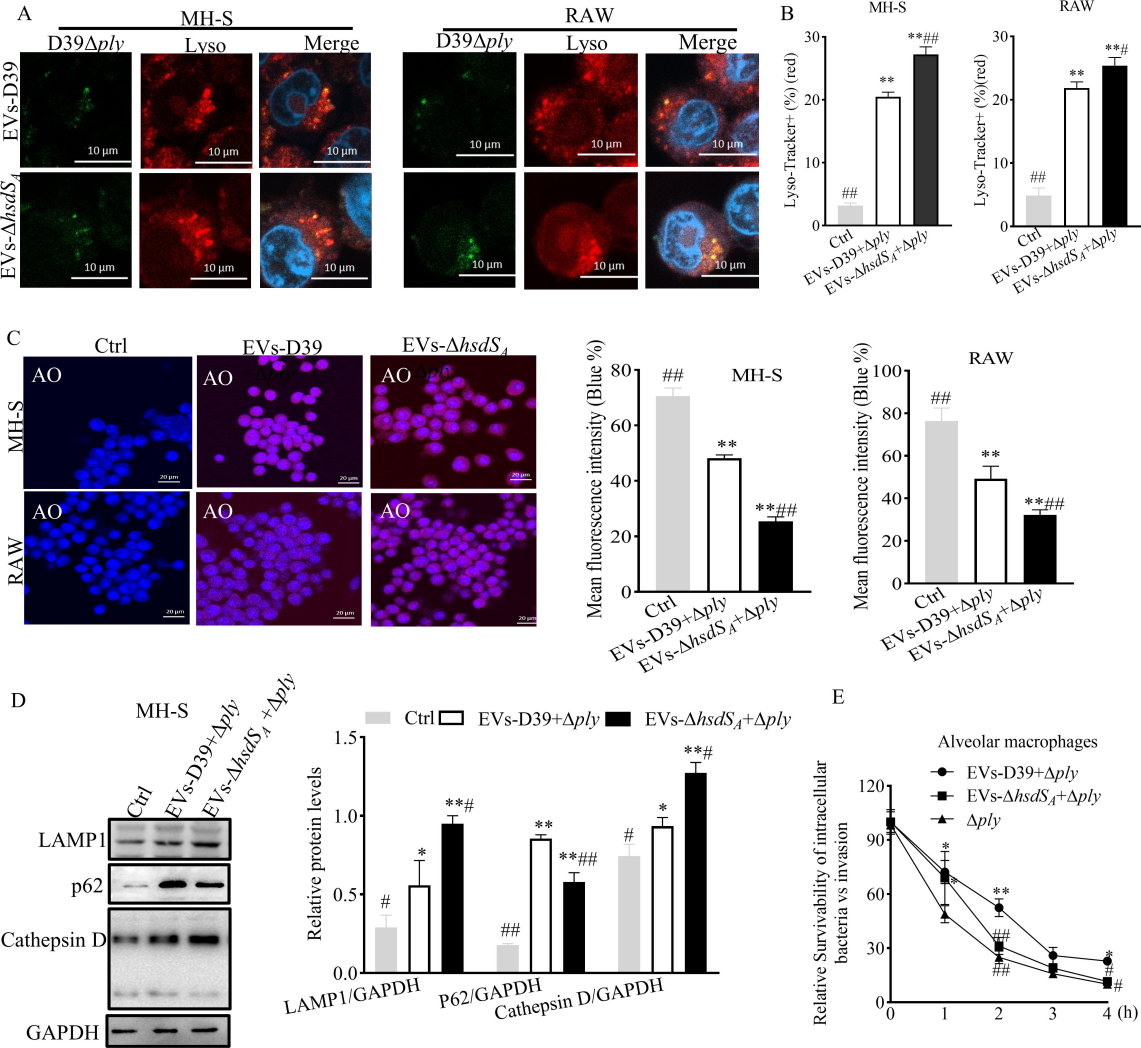
EV-associated PLY reduced lysosome acidification to evade host immune defenses against *S. pneumoniae*. Macrophages were stimulated with EVs for 4 h and then infected with D39Δ*ply* for 2 h. (**A**) Confocal microcopy for the co-localization of intracellular bacteria with LysoTracker-labeled lysosomes. Cells were photographed after extracellular bacteria killing by antibiotic and another 3 h of continuous culture. Green indicates FITC-labeled *S. pneumoniae*; red denotes LysoTracker-stained lysosome. (**B**) Quantification of LysoTracker fluorescence intensity. (**C**) AO staining for lysosomal membrane permeability. Blue denotes the binding of AO to cytoplasmic and nucleolar RNAs; red indicates the accumulation of AO in acidic vesicles such as lysosomes. (Right panel) Quantification of blue fluorescence intensity. (**D**) Western blot for the expression of LAMP1, p62, and cathepsin D. (**E**) EVs affect the intracellular survival of D39Δ*ply* in mouse alveolar macrophages under EV pre-treatment. Statistical analysis was analyzed by an unpaired Student *t*-test and performed using Prism (version 8.0, EVs-D39+Δ*ply* vs EVs-D39Δ*hsdS_A_* +Δ*ply*. *P<0.05, **P<0.01. All statistical analyses were analyzed by one-way ANOVA followed by Dunnett’s multiple comparison. *P<0.05, **P<0.01 vs Ctrl; ^#^ *P* < 0.05, ^##^ *P* < 0.01 vs EVs-D39 + D39Δ*ply*.

Interestingly, maturation of LAPosomes containing pathogenic bacteria is a complex process during host killing bacteria. Some pathogens target molecules involved in LAP formation through virulence factors, hiding in non-acidic LAPosomes to prevent recognition by xenophagy. Although *Listeria* hemolysin O (LLO) and streptococcal hemolysin O (SLO) both induce macrophage LAP formation, they can form pores on the LAPosome membrane to contribute to the insufficient LAPosome acidification, leading to intracellular survival of bacteria in non-degradable LAPosomes (18, 20). As PLY is a member of bacterial pore-forming toxins, we thought that it might share a similar mode of action as LLO and SLO to help *S. pneumoniae* against macrophage elimination. To investigate this hypothesis, macrophages were infected with fluorescein isothiocyanate (FITC)-labeled D39Δ*ply* for 2 h, followed by extracellular bacteria clearance and 4 h of continuous culture. Confocal microcopy was used to observe the co-localization of intracellular bacteria with LysoTracker-labeled lysosomes. Accordingly, under EVs-D39 pre-treatment, the co-localization between D39Δ*ply* and the Red LysoTracker-labeled lysosomes was significantly decreased compared with EVs-D39Δ*hsdS_A_* pre-treatment (Fig. 7A and B), while it was found in Fig. 5A that the intracellular bacterial loads were increased, indicating that the intracellular bacteria had escaped from vacuoles into the cytoplasm. For this purpose, acridine orange (AO), which emits blue fluorescence after binding to cytoplasmic and nucleolar RNAs but red fluorescence after accumulating in acidic vesicles such as lysosomes (37), was used to evaluate the lysosome membrane integrity at this time point. Indeed, under EVs-D39 pre-treatment, the mean blue fluorescence intensity of AO was 1.9- and 1.5-fold greater than that of EVs-D39Δ*hsdS_A_* pre-treated MH-S and RAW cells, respectively (Fig. 7C), indicating a better integrity of lysosome membrane. This suggests that a high concentration of PLY enriched in the EVs-D39 could damage the integrity of lysosome membrane, which was similar to the reported LLO and SLO. Moreover, the expression levels of lysosome membrane protein (LAMP1), p62, and lysosomal hydrolase cathepsin D were also detected. Notably, in the EVs-D39 pre-treatment group, the expression of LAMP1 and cathepsin D were much lower; however, the expression of p62 was higher (Fig. 7D; Fig. S6D). These data indicated that EVs were beneficial for the fusion between LAP and lysosomes but increased the permeability of lysosome membrane, which resulted in H^+^ loss inducing an elevated pH gradient. Finally, bacteria evade against the lysosome’s defenses. To further verify this conclusion, we extracted mouse alveolar macrophages and counted the number of intracellular bacteria at each time point after infection. Similar to the previous results, EV pre-treatment prolonged the survival time of D39Δ*ply* in macrophage cells, especially for EVs-D39, with a prolonged time that was twofold higher than that for EVs-D39Δ*hsdS_A_* (Fig. 7E).

Together, the data above indicate that EV-associated PLY promoted the fusion of LAP and lysosome by destroying the integrity of the lysosome membrane and leading to the failure of lysosome acidification and *S. pneumoniae* elimination.

### EV-associated PLY mediated *S. pneumoniae* to evasion of immune defenses in mice

**Fig 8:**
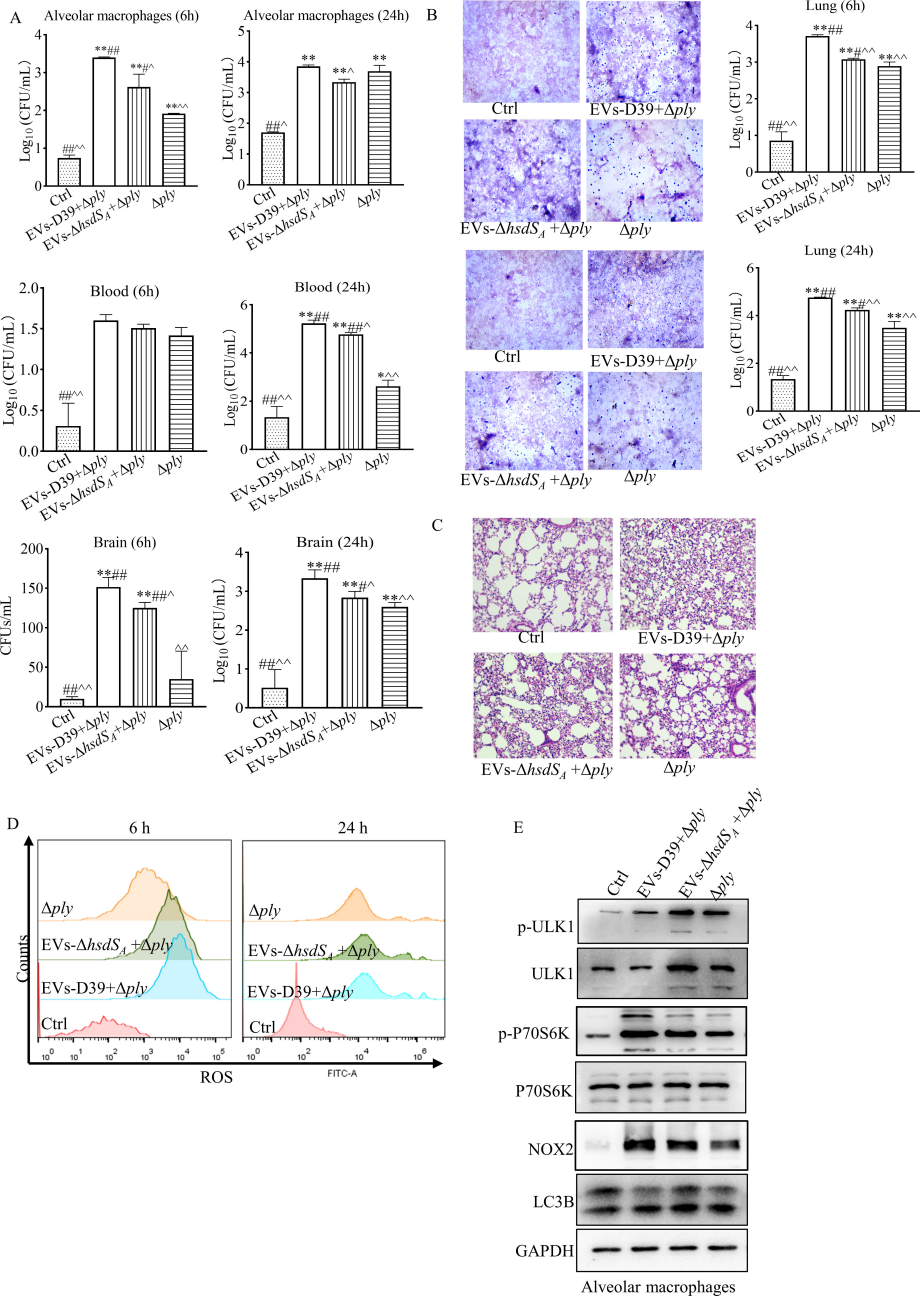
EV-associated PLY mediates *S. pneumoniae* evasion of immune defenses in mice. Mice were pre-treated with 100-µg EVs by intranasal implantation for 4 h, followed by D39Δ*pl*y intranasal infection with 8.5 × 10^8^ CFU for 6 and 24 h, respectively. (**A and B**) Plate count methods and Gram staining for bacterial loads in mouse alveolar macrophages, blood, lung, and brain tissues at 6 and 24 h after infection. (**C**) Hematoxylin and eosin staining of mouse lung tissues. (**D**) Representative flow cytometry analysis of ROS release in mouse alveolar macrophages. (**E**) Western blot for the expression of LC3B, NOX2, ULK1, p-ULK1, P70S6K, and p-P70S6K in mouse alveolar macrophages. All statistical analyses were analyzed by one-way ANOVA followed by Dunnett’s multiple comparison. *P<0.05, **P<0.01 vs Ctrl; ^#^ *P* < 0.05, ^##^ *P* < 0.01 vs Δ*ply*; ^^^ *P* < 0.05, ^^^^ *P* < 0.01 vs EVs-D39 + D39Δ*ply*.

Next, to further confirm the role of EV-associated PLY in *S. pneumoniae* evasion of host immune defense, mice were pre-treated with 100-µg EVs by intranasal implantation for 4 h, followed by D39Δ*pl*y intranasally infected with 8.5 × 10^8^ CFU for 6 and 24 h, respectively. BALF was collected for alveolar macrophage extraction. Blood, lung tissue, and brain tissue of mice were taken at each time point. Results from plate count methods and Gram staining showed that during invasive infection, bacterial loads of alveolar macrophages, brain, and lung in the EV pre-treatment group were significantly higher than that in only D39Δ*ply* infection group at 6 h, and similar results were observed in blood, brain, and lung at 24 h. More importantly, bacterial loads in alveolar macrophages and lung tissue pre-treated with EVs-D39 were much higher, compared to the EVs-D39Δ*hsdS_A_* pre-treatment group at both 6 and 24 h. Meanwhile, bacterial loads in blood and brain tissue of EVs-D39 pre-treated mice were also highest at 24 h after infection (Fig. 8A and B). Consequently, mouse lung tissues in the EVs-D39 pre-treatment group displayed the most severe pulmonary edema and inflammatory invasion (Fig. 8C).

Moreover, the extracted alveolar macrophages from different groups were subjected to a flow cytometry assay for ROS production. Similar to the *in vitro* data, EVs-D39 pre-treatment resulted in a more significant increase in ROS production at 6 h after infection; however, this difference disappeared at 24 h (Fig. 8D). Furthermore, we also detected the expression levels of LC3B, NOX2, ULK1, and P70S6K in mouse alveolar macrophages at 6 h after infection. As expected, LC3BI/II conversion occurred in all the infected groups, and the dramatically increased expression of NOX2 but decreased expression of p-ULK1 were found in the EVs-D39 pre-treatment group. In contrast, ULK1 phosphorylation was increased in the EVs-D39Δ*hsdS_A_* pre-treatment group (Fig. 8E; Fig. S7), indicating that EV-associated PLY was conducive for *S. pneumoniae*-induced LAP formation in mouse alveolar macrophages to evade immune defense.

## DISCUSSION

To date, some elements that affect the phase variation are largely missing. In *S. pneumoniae*, the potential of type I restriction modification (RM) system has been deemed to mediate epigenetic changes via gene regulation (38, 39). The type I RM systems contain three co-transcribed genes (*hsdR*, *hsdM*, and *hsdS_A_*), a separately transcribed Cre tryrosine DNA recombinase gene, and two truncated *hsdS* genes (*hsdS_B_* and *hsdS_C_*) (40). In this study, we found that the knockout of *hsdS_A_* in the *S. pneumoniae* D39 strain caused an obvious change in colony phase and an unexpected decrease in capsule production. These results were in agreement with previous studies of the transcriptional repressor FabT mutation (41). Importantly, the knockout of *hsdS_A_* did not affect *S. pneumoniae* growth but increased mRNA expression of PGN synthesis-related genes, followed by an elevated PGN level and cell wall thickness. Ulteriorly, we confirmed that *hsdS_A_* knockout was most likely responsible for the enhanced adhesion of *S. pneumoniae* to epithelial cells and the reduced anti-phagocytic capacity in macrophages. Moreover, *ΔhsdS_A_* also led to a decreased virulence of *S. pneumoniae* challenged mice, which may mainly be attributed to the decrease of CPS but the increase of PGN. Interestingly, we also found that *hsdS_A_* knockout prolonged the survival of *S. pneumoniae* in several types of macrophages, indicating that the immune escape ability of bacteria was increased.

MVs produced by Gram-positive bacteria activate pattern recognition receptors (PRRs) to mediate pathogenesis and cause disease in the host. For example, upon interaction with host epithelial cells, MVs are detected by the membrane receptor TLR2 and subsequently enter into host cells to facilitate their interaction with the endosomal receptors for nucleic acids, TLR7, TLR8, and TLR9, and the cytoplasmic receptor for peptidoglycan, NOD2, leading to the production of pro-inflammatory cytokines and chemokines. The intracellular detection of MVs triggers the formation of autophagosomes and the processing of MVs via the host cellular degradation pathway of autophagy to ultimately facilitate their clearance from the host (27). Importantly, it has been reported that the thick peptidoglycan layer is one of the main factors affecting the release of EVs from Gram-positive bacteria (13). Here, the transparent colonies generated by *ΔhsdS_A_* had significantly increased PGN contents, suggesting that the occurrence of phase variation may affect the release of EVs. To unravel the role of *hsdS_A_* in *S. pneumoniae* EV production and the role of EVs in pneumococcal escape from host immunity, we isolated the EVs and found that EVs obtained from D39 displayed higher protein concentrations than those from D39Δ*hsdS_A_* at the same amounts of bacteria, indicating that *hsdS_A_* knockout reduced MV release. Attentively, there was a markedly differential protein A between 50 and 70 kDa. Combined with quantitative proteomic analysis, we determined that protein A was PLY. Microbial EVs encapsulate cargo that includes lipids, proteins, and nucleic acids, which have been shown to play roles in microbial physiology, pathogenesis, and the transmission of biological signals into host cells to modulate biological processes and host innate immune responses (42, 43). Furthermore, PLY was found to be predominantly contained in EVs, and PLY in EVs-D39 PLY was significantly higher than that in EVs-D39Δ*hsdS_A_*. Surprisingly, compared with the EV pre-treatment group, D39Δ*ply* strain had significantly reduced survival ability in macrophages, particularly at later time points of 3 and 4 h. More importantly, EVs-D39 pre-treatment was more conducive to the intracellular survival of *S. pneumoniae* than EVs-D39Δ*hsdS_A_* pre-treatment, indicating that the survival of *S. pneumoniae* in macrophages was closely related to EV-associated PLY.

Previous studies have shown that many pathogens have evolved strategies to promote their survival and proliferation, either by targeting molecules in the LAP pathway with virulence factors or by hiding in LAPosomes to prevent recognition by cytoplasmic surveillance mechanisms, such as xenophagy. For example, *Listeria monocytogenes* replicates in macrophage vacuoles by LLO and inhibits LAP by modulating mitochondrial Ca^2+^ signaling (20, 44). Group A streptococcus infection preferentially induces ineffective LAP to evade Xenophagic killing in endothelial cells through the SLO activated NOX2 pathway (18). Importantly, PLY, along with SLO and LLO, belongs to the cholesterol-dependent cytolysin family, which is of broad importance to a diverse group of pathogens (45). LAP, such a non-canonical autophagy pathway, is induced by activation of specific surface receptors of phagocytes and results in formation of so-called LAPosome-decorated single-membrane vesicles with LC3 (46). LAPosomes are a process that strictly depends on ROS produced by the NADPH oxidase CYBB/NOX2 (17). In contrast, the ULK complex is required for initiation of canonical forms of autophagy such as xenophagy, which possesses a characteristic double-membrane vesicle (47). Consequently, we found that under EVs-D39 pre-treatment, the majority of monolayer structure surrounding the D39Δ*ply* appeared in the macrophages; however, under EVs-D39Δ*hsdS_A_* pre-treatment, a large amount of double-layered structure appeared in the macrophages around the D39Δ*ply* instead of the single-layered structure. Furthermore, ROS release, NOX2 puncta co-localized with LC3, and p-P70S6K expression in EVs-D39 pre-treatment group were significantly higher than those in the EVs-D39Δ*hsdS_A_* pre-treatment group. However, the p-ULK1 expression levels of in EVs-D39Δ*hsdS_A_* pre-treatment were more obvious. Thus, it seems conceivable that EV-associated PLY was conducive for macrophages to target *S. pneumoniae* by LAP. To explore which receptor binds PLY to activate LAP, we examined the expression of integrin β1, β2, and aVβ3 and found that there was a significant difference in the expression of integrin β1 and aVβ3. However, aVβ3 has been implicated in treatment of tumors (48, 49). Therefore, we determined that EV-associated PLY could induce NOX2 and LAP activation via β1 integrin receptor.

LLO can block nascent phagosome maturation by uncoupling the pH gradient across the phagosomal membrane (20). SLO induces LAP formation by promoting β1 integrin expression and leads to insufficient LAPosome acidification, which is beneficial to intracellular proliferation of group A streptococcus (18). Of note, PLY is homologous to LLO and SLO and may play a similar role in damage to the LAPosomal membrane and uncouple pH gradients in macrophage. In fact, we observed the co-localization between D39Δ*ply* and lysosome was significantly decreased in the EVs-D39 pre-treatment group, compared with the EVs-D39Δ*hsdS_A_* pre-treatment group. In addition, integrity of lysosome membrane and that expression of lysosomal hydrolase cathepsin D and LAMP1 after pre-treatment with EVs-D39Δ*hsdS_A_* were significantly enhanced, indicating that EV-associated PLY promoted LAP fusion with lysosomes but disrupted the integrity of the lysosome membrane, which in turn altered the pH gradient, resulting in the loss of hydrolase activity and ultimately bacterial escape from the host cell. Finally, we extracted mouse alveolar macrophages to further confirm the above intracellular survival results and reached the same conclusion.

**Fig 9:**
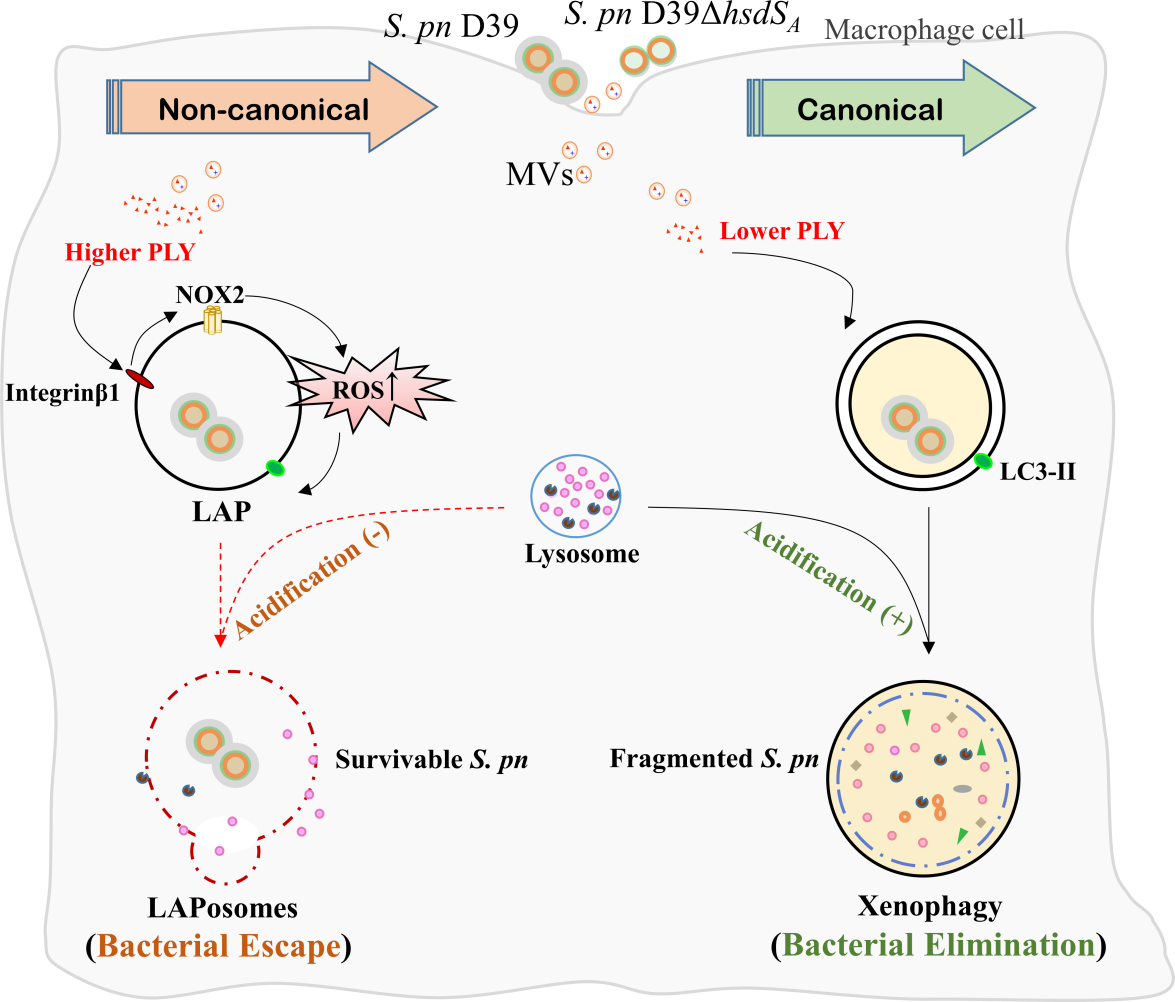
Model depicting variable intracellular fates of *S. pneumoniae* due to *hsdS_A_* regulated extracellular vesicle-associated PLY during infection of macrophages. *S. pneumoniae* and EVs- associated PLY were internalized into macrophages to induce LC3II conversion. Higher EV-associated PLY (produced by D39) upregulated β1 integrin, followed by NOX2 activation and ROS production for LAP formation, which increased the permeability of lysosome membrane and induced an insufficient acidification to escape the host killing. In contrast, lower EV-associated PLY (produced by D39Δ*hsdS_A_*) activated ULK1 recruitment to form double-layered autophagosomes to eliminate bacteria.

In conclusion, our study confirmed a gene related to colony transparency of *S. pneumoniae*, *hsdS_A_*, which plays a very important role in virulence. Most importantly, *hsdS_A_* knockout reduced the production of EVs, affecting PLY contents in EVs. EV-associated PLY induced LAP formation by activating the β1 integrin/NOX2/ROS pathway, promoting LAPosome fusion with lysosome. At the same time, EV-associated PLY damaged the integrity of lysosome membrane and changed the pH gradient, resulting in the lysosome being unable to lyse intracellular *S. pneumoniae*, and ultimately prolonging the survival of *S. pneumoniae* in macrophages (Fig. 9).

## MATERIALS AND METHODS

### Bacterial culture and and constructs

*S. pneumoniae* strain D39 (NCTC 7466, serotype 2) and strain TH7898 containing Janus casette were both donated by Professor Jingren Zhang of Tsinghua University (44). Erythromycin-resistant *S. pneumoniae* CPM8 and plasmid pJWV25 were donated by Professor Xuemei Zhang of Chongqing Medical University (37). *Escherichia coli* DH5α was purchased from Biyuntian Bio. All *S. pneumoniae* strains were grown as standing cultures in Todd-Hewitt broth (Meilunbio, Liaoning, China) supplemented with 0.5% yeast extract (Meilunbio) (THY) broth, or Columbia agar plates (SenBeiJiabio, Nanjing, China) with 5% sheep blood (Meilunbio) at 37°C in a 5% CO_2_ in air atmosphere.

The construction of pneumococcal knockout was carried out in the paternal D39, a streptomycin-resistant derivative of strain D39 (50). In brief, the upstream and downstream sequences of the *hsdS_A_* intergenic region were amplified with primer P5/P6 and P7/P8 from the wild D39, and the Janus cassette (referred to as JC) was amplified with P9/P10 from the strain TH7457 (9). The overlap extension fusion PCR was performed with P5/P9 and P7/P10 before being transformed into D39 to generate D39Δ*hsdS_A_*::JC. The upstream and downstream sequences of the *hsdS_A_* region were fused by overlap primer P13/P14. The fusion PCR product was transformed into D39Δ*hsdS_A_*::JC to generate unmarked D39Δ*hsdS_A_* knockout. To construct the *ply* knockout strain (D39Δ*ply*), the upstream and downstream sequences of the *ply* intergenic region were amplified with primers P15/P16 and P19/P20 from the wild D39, and the erm cassette was amplified with P17/P18 from the strain CMP8 (51). The overlap extension fusion PCR was performed with P15/P18 and P17/P20 before being transformed into D39 to generate D39Δ*ply* using the primers listed in Table S1.

### Cell cultures

Epithelial cells, including mouse non-epithelial cells (A549), and human bronchial epithelial cells (Beas-2B) and macrophages, including mouse alveolar macrophages (MH-S) and mouse peritoneal macrophages (RAW264.7), were cultured in Dulbecco’s modified Eagle medium (DMEM; Gibco, New York, USA) supplemented with 10% fetal bovine serum (FBS) (Gibco), 100-µg/mL gentamicin (Sigma, Shanghai, China), and 60-µg/mL kanamycin (Sigma) at 37°C in a 5% CO_2_ in air atmosphere.

### Growth curve

*S. pneumoniae* was prepared by taking 100-µL overnight cultured frozen stock to 5 mL of fresh THY medium with the corresponding antibiotic and placing it at a 37°C (5% CO_2_) incubator for static culture. The absorbance values (OD_600_) of bacterial cultures were measured every 1 h with 13 h time periods. Triplicate absorbance readings were compared with time to generate bacterial growth curves.

### Pneumococcal colony opacity observation

Stereoscopic observation of pneumococcal colonies for opacity was performed using TSA plates supplemented with catalase (Sigma) as previously described (9, 41). Briefly, 50 µL of bacterial solution diluted with PBS to approximately 10^4^ CFU/mL was mixed with 100-µL of catalase (20 mg/mL) and spread evenly on the prepared TSA solid culture plates. The colony opacity of bacteria on each TSA plate was observed with a stereo microscope after the plates were incubated in a 5% CO_2_ incubator at 37°C for 24 h. Each strain was repeated three times in parallel.

### TEM

Bacteria were cultured as described above. The bacterial clusters were fixed with 2.5% glutaraldehyde for 24 h and embedded into 2% agarose, and processed by the Electron Microscopy Research Service of China Pharmaceutical University, and the thickness of bacterial cell wall was quantified by measuring 15 randomly chosen cells using Image-Pro Plus (version 6.0).

### Quantification of CPS and PGN

Quantification of *S. pneumoniae* CPS was conducted by the uronic acids as described in references (52, 53), whereas PGN was assayed according to the instructions of the Bacteria PGN ELISA kit (Shinoda Biotechnology, China). Briefly, the 150-mM Tris-HCl, pH7.0, and 1-mM MgSO_4_ resuspended samples were incubated with 0.01% deoxycholate at 37°C for 30 min to lyse the cells. Each sample was added to 600-µL 12.5-mM sodium tetraborate in 98% H_2_SO_4_, vortexed, and heated at 95°C for 5 min. Then with the addition of 3-phenylphenol solution, the colors of the samples cooled on ice were observed immediately, or the whole cell CPS was quantified by reading the absorbance value at 495 nm in a 96-well plate. The results of representative experiments are presented as the means of three replicates ± standard deviations. Each sample was processed three times in parallel.

### Reverse transcription-quantitative real-time PCR (qRT-PCR)

The total RNA rapid extraction reagent of Yifeixue BioTech was employed for total bacterial RNA extraction according to the manufacturer’s instructions. HiScript Q RT SuperMix was used for reversal reaction from total RNA according to the manufacturer’s instructions. Then real-time fluorescence quantitative analysis was carried out by ChamQ Universal SYBR qPCR Master Mix. The representative experimental results are presented as the means of three replicates ± standard deviations.

### Cell viability/cytotoxicity assay

MH-S and RAW264.7 cells were seeded per well in a 12-well plate and allowed to adhere overnight. Cells were infected with pneumococcal suspension at MOI = 40 or 20 and incubated at 37°C for 3 h. After infection, the effects of different MOIs on cell viability or cytotoxicity were evaluated according to the instructions of the calcein/propidium iodide (PI) cell viability/cytotoxicity assay kit (Beyotime).

### Adhesion assay

Adhesion assay was performed as described previously (54). To test the adhesion of D39 and D39Δ*hsdS_A_*, Beas-2B and A549 were seeded into 12-well plates at a density of 5 × 10^5^ cells per well and allowed to grow for 24 h. Cells were infected with pneumococcal suspension containing 1 × 10^7^ CFU/mL, resulting in an MOI of 20 and incubated at 37°C for 2 h. After infection, the cells digested from the well plate were lysed with 0.01%TritonX-100 for 5 min. The above-mentioned cell lysate was diluted with THY liquid medium and then spread on a Columbia plate supplemented with 5% sheep blood and placed in a CO_2_ incubator at 37°C for 18 h. The overnight cultured plate was taken and the number of colonies of different strains was counted on the blood plate. Three sets of experiments were performed in parallel for each experiment.

### Phagocytosis assay

Similarly, phagocytosis assay was performed as described previously (55, 56). MH-S and RAW264.7 cells were seeded per well in a 12-well plate and allowed to adhere overnight. Cells were infected with pneumococcal suspension at MOI = 20 and incubated at 37°C for 2 h. Cells were incubated for 60 min in DMEM culture medium containing 10-µg/mL penicillin and 200-µg/mL gentamicin to kill extracellular pneumococci. Intracellular bacteria were counted as above. Three sets of experiments were performed in parallel for each experiment.

### Penicillin gentamicin protection assay

Infection assays were performed as described earlier (29). Briefly, MH-S and RAW264.7 were infected at an MOI of 20 (for D39, D39Δ*hsdS_A_*, and D39Δ*ply* strains) for 2 h and further incubated in culture medium containing penicillin (10 µg/mL) and gentamicin (200 µg/mL) for 2 h to kill the extracellular bacteria. Cells were lysed (ddH_2_O), and serial dilutions of the lysates were plated on Columbia agar plates with 5% sheep blood for enumeration of bacterial colonies. To assess intracellular survival trend of bacteria, cell lysates were prepared similarly and spread plated at indicated time intervals following 1 h. Surviving bacteria at different time points were enumerated and were represented as percent survival at indicated time points relative to 0 h (post antibiotic treatment). For EVs studies, macrophages were pre-treated with 50-µg/mL EVs-D39 or EVs-D39Δ*hsdS*_*A*_ for 4 h before infection with D39Δ*ply* strain.

### Isolation and purification of EVs

Isolation and purification of EVs were prepared as previously described (13). *S. pneumoniae* logarithmic phase supernatant was filtered and concentrated with a 100-kDa tangential flow filtration system (Pall Corporation). The retentate was filtered again before centrifugation at 150,000 × *g* for 3 h at 4°C to pellet the vesicles and leave soluble proteins in the supernatant. Each fraction was subjected to SDS-PAGE and stained with a Coomassie brilliant blue. EV samples were evaluated with a Nanobrook ZetaPALS potential analyzer (Brookhaven Instruments Corporation) and visualized by TEM. Finally, quantitative proteomic analysis was performed by Biotech Pack.

### EV cytotoxicity

Macrophages were pre-treated with 50 µg/mL for 6, 12, and 24 h. Cell viability was assessed using MTT [3-(4,5-dimethylthiazol-2-yl)-2,5-diphenyltetrazolium bromide] assay kit (Sigma) according to the manufacturer’s protocol. Untreated cells were used as negative control, while Triton X-100 (0.025%) was used as positive control. The data are from two independent experiments, each one performed in triplicate.

### Hemolysis assay

Hemolysis assay was performed by adopting the protocol as described earlier (25). Briefly, different concentrations of EVs or other components were mixed with an equal volume of 2% RBC and incubated at 37°C for 60 min. After removing undissolved RBCs by centrifugation (1,500 rpm, 10 min), 100 µL of supernatant was removed to a 96-well plate. Absorbance of the released hemoglobin in the supernatants was determined at 450 nm using a microplate spectrophotometer (Thermo Fisher Scientific). PBS and Triton X-100 (0.1%) were used as negative and positive controls, respectively. Hemolysis rate (%) = (EV group OD450 − negative control OD450) / (positive control OD450 − negative control OD450) × 100%.

### Identification of protein composition in EVs by proteomics

The principle of proteomics is that the same peptides ionize with the same efficiency in mass spectrometry analysis. The area can directly represent the amount of the peptide, so it can be directly obtained by comparing the peak area of the mass spectrum. Relative quantitative results of the proteins were represented by this peptide. Comparative proteomic analysis was performed on the two kinds of vesicle samples. The samples were first reduced, alkylated, and then subjected to trypsin digestion, after which the processed samples were prepared. The samples were analyzed by liquid chromatography-tandem mass spectrometry, and the raw results of the mass spectrometry were obtained in the raw file, which was processed by the software MaxQuant (version 1.6.2.10) analysis, matching data, and obtaining the results of identification of differential proteins.

### Flow cytometry

For flow cytometry, EVs were labeled with DiO and pre-treated with 50 µg/mL for 3 and 6 h, washed, and analyzed with flow cytometer using FITC channel. During pre-treatment with 50 µg/mL for 24 h, apoptosis was detected by an apoptosis kit (Beyotime, Shanghai, China). After *S. pneumoniae* strain infection as described above, cells were collected and then coincubated with a ROS Assay Kit (Beyotime). Data were processed and interpreted using FlowJo software. The data are from two independent experiments, each one performed in triplicate.

### PLY content assay

Using BCA kit to adjust the concentration of EVs to 30, 50, and 100 µg/mL, we operated the ELISA kits (Shanghai Panco) to detect PLY content in EVs at each concentration. After stimulation of cells with 50-µg/mL EVs for 6 h, the supernatant was discarded and washed three times with PBS, and the intracellular PLY content was assessed according to the PLY ELISA kit instructions.

### Confocal imaging

To observe the internalization, EVs were labeled with DiO, whereas cell membranes were labeled with DiD. Macrophages was pre-treat with 50 µg/mL for 2 h and 6 h, washed and imaged with confocal microscopy. To assess co-localization of LC3 with NOX2 or ULK1, macrophages were infected with D39Δ*ply* for 2 h before EVs pre-treatment for 4 h and then fixed with 4% paraformaldehyde (Santa Cruz, Shanghai, China) for 30 min at room temperature (RT), permeabilized with 0.1% Triton X-100 (Sigma-Aldrich) for 30 min at RT, blocked with 1% bovine serum albumin (BSA; Sigma-Aldrich) for 30 min at RT, and stained with anti-LC3 (Santa Cruz), anti-NOX2 (Cell Signaling Technology, Shanghai, China), anti-ULK1 (Cell Signaling Technology) at 4°C overnight. After the cells were washed three times with PBS, they were stained with Alexa Fluor-conjugated secondary antibodies (Invitrogen, Shanghai, China) and 2-(4-amidinophenyl)-6-indolecarbamidine dihydrochloride (Beyotime) for nuclear staining for 30 min. To assess intracellular survival trend of bacteria and lysosome, LysoTracker (red DND-99; Invitrogen), an acid indicator staining, was added at a final concentration of 200 nM in medium for 1 h of incubation at 37°C in the dark. All samples were performed using a Carl Zeiss LSM 800 confocal microscope, and the images were analyzed using ZEN Black software. The data are from two independent experiments, each one performed in triplicate.

### Mouse infection assays

Mouse infection assays were performed as previously described (57, 58). All the animal experiments were discussed with and approved by the Animal Care and Use Committee of China Pharmaceutical University. Animals conformed to animal protection laws of the People’s Republic of China and applicable guidelines. To evaluate the effect of *hsdS_A_* on the virulence of *S. pneumoniae*, we constructed a mouse intranasal infection model with 1 × 10^8^ CFU of D39 and D39Δ*hsdS_A_* in 4-week-old-ICR mice (each group consisted of 12 animals). Mouse survival was monitored daily for 7 days with each group consisted of 6 animals. To determine the organ involvement, blood and BALF were collected from mice at 24 h after infection. Lung and brain tissues were homogenized. Bacterial counts and Gram stain in the BALF as well as lung homogenates were determined by separately plating serial dilutions and microscope. IL-6 and TNF-α in the lung homogenates were analyzed by ELISA kits (MULTI Sciences).

To further confirm the role of EV-associated PLY in *S. pneumoniae* evasion of host immune defense, mice were pre-treated with 100-µg EVs by intranasal implantation for 4 h, followed by D39Δ*pl*y intranasally infected with 5 × 10^8^ CFU for 6 and 24 h, respectively. Bronchial lavage fluid used to extract alveolar macrophages, blood, lung tissue, and brain tissue of mice was taken at each time point for plate count of bacteria load. In addition to these, the lung tissue was also stained with Gram staining and hematoxylin and eosin examination to further evaluate the bacterial load and pathological damage (the general indexes of pulmonary edema, congestion and inflammatory cell infiltration in the pulmonary interstitium and alveoli).

### Lysosomal membrane permeability assay

Lysosomal membrane permeability assay was prepared for experiments as previously described in more detail (37, 59). In order to observe intracellular lysosome membrane permeability change, 2-µg/mL acridine orange (Sigma) was added to the cells infected with D39Δ*ply* for 2 h. Then, the slides were cultured for another 15 min with 5% CO_2_ at 37°C, washed with PBS, and fixed by 4% paraformaldehyde. Confocal microscopy was used to observe intracellular fluorescence intensity changes, and images were collected.

### Western blotting

Cell samples were then subjected to SDS-PAGE and proteins were transferred to polyvinylidene difluoride (PVDF) membranes (Millipore). After blocking with 5% skim milk or BSA in TBST, the membranes were incubated overnight at 4°C with primary antibodies, including anti-PLY, anti-LC3B, anti-NOX2, anti-p70s6k antibody, anti-phospho-p70s6k antibody (Thr389), anti-ULK1 antibody, anti-phospho-ULK1 antibody (Ser555), anti-β1 integrin antibody, anti-β2 integrin, anti-αVβ3 integrin, anti-GAPDH antibody, and anti-actin antibody. The above-named antibodies were purchased from Santa Cruz. After incubation with rabbit-conjugated secondary antibody (Cell signaling Technology) at RT for 2 h, membranes were soaked in ECL solution, and the images were captured by a luminescence imaging system.

### Statistical analysis

Data obtained from independent experiments are presented as the means ± standard deviations. Comparisons of two sets of the data were analyzed by an unpaired Student *t*-test. For three or more sets of data, statistics were analyzed by one-way analysis of variance with Tukey’s multiple comparisons post test. Statistical analysis was performed using SPSS (version 18.0) software, and data processing was performed using Prism (version 8.0), respectively. A *P* value of < 0.05 or <0.01 was considered statistically significant.

## SUPPLEMENTAL MATERIAL

Supplemental material spectrum.00995-23-s0001.docx Table S1, Figure S1, Figure S2, Figure S3, Figure S4, Figure S5, Figure S6, Figure S7.

ASM does not own the copyrights to Supplemental Material that may be linked to, or accessed through, an article. The authors have granted ASM a non-exclusive, world-wide license to publish the Supplemental Material files. Please contact the corresponding author directly for reuse.
