# Supplementary material for: hsdSA regulated extracellular vesicle-associated PLY to protect Streptococcus pneumoniae from macrophage killing via LAPosomes
Source: Microbiol Spectr. 2023 Nov 29;12(1):e00995-23. doi: 10.1128/spectrum.00995-23 (PMC10783081; doi:10.1128/spectrum.00995-23)
Supplement: Supplemental material — Table S1, Figure S1, Figure S2, Figure S3, Figure S4, Figure S5, Figure S6, Figure S7. [file spectrum.00995-23-s0001.docx]

| **Primer** | **Sequence (5´-3´)** | **Template** | **Amplicon Product** | **Size (bp)** |
| --- | --- | --- | --- | --- |
| P1 | gacgtgctgacaaatgttgcaa | D39 genome | rpsl-UP | 287 |
| P2 | gctgagttaggtgttttaggtgtca |  |  |  |
| P3 | tgacacctacaaaacctaactcagc | D39 genome | rpsl-DOWN | 397 |
| P4 | tacgtcacgttttggagctct |  |  |  |
| P5 | taaattagtgcgtcaatatgggcg | D39 genome | *hsdS_A_* | 1569 |
| P6 | caccagaacaacttaaagcaagta |  |  |  |
| P7 | tgttgttcagtagctgttttccttag | D39 genome | *hsdS_A_*-UP | 824 |
| P8 | ccttcaaggagttttcagcattatccgtataatagttttacgtgcggtgga |  |  |  |
| P9 | tccaccgcacgtaaaactattatacggataatgctgaaaactccttgaagg | HT7898 genome | JC | 1459 |
| P10 | gcagttgttggaggacaaataatgacctttccttatgcttttggacgttt |  |  |  |
| P11 | aaacgtccaaaagcataaggaaaggtcattatttgtcctccaacaactgc | D39 genome | *hsdS_A_*-DOWN | 917 |
| P12 | aacttggagaatcaatggcagac |  |  |  |
| P13 | gcagttgttggaggacaaataatgagtataatagttttacgtgcggtgga | *hsdSA*-UP,  *hsdSA*-DOWN | *hsdS_A_*-UP-DOWN | 1741 |
| P14 | tccaccgcacgtaaaactattatactcattatttgtcctccaacaactgc |  |  |  |
| P15 | gctttctggagtttcccgattt | D39 genome | *ply*-UP | 825 |
| P16 | ATCAAACAAATTTTgggcccggcttctacctcctaataagttcctgga |  |  |  |
| P17 | tccaggaacttattaggaggtagaagccgggcccAAAATTTGTTTGAT | CPM8 | erm | 859 |
| P18 | tgtcgcaagcattctcctctcagtcggcagcgactcatagaat |  |  |  |
| P19 | attctatgagtcgctgccgactgagaggagaatgcttgcgaca | D39 genome | *ply*-DOWN | 941 |
| P20 | tagagttggctccatctttagc |  |  |  |

Table1 Primer sequences for strain constructs


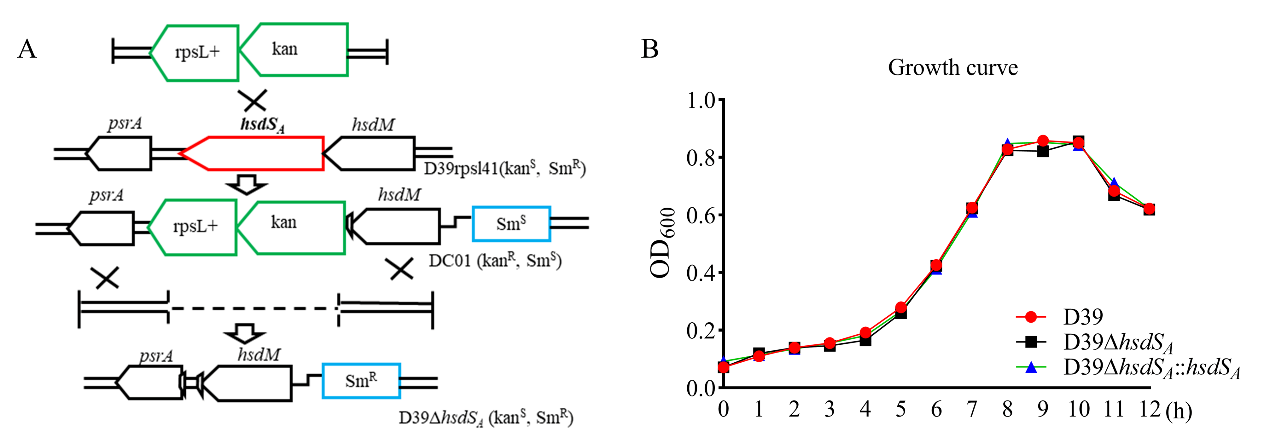
Figure S1 Effect of *hsdS_A_* knockout on growth of *S. pneumoniae*. (A) Construction of the knockout strain. (B) Growth curve.


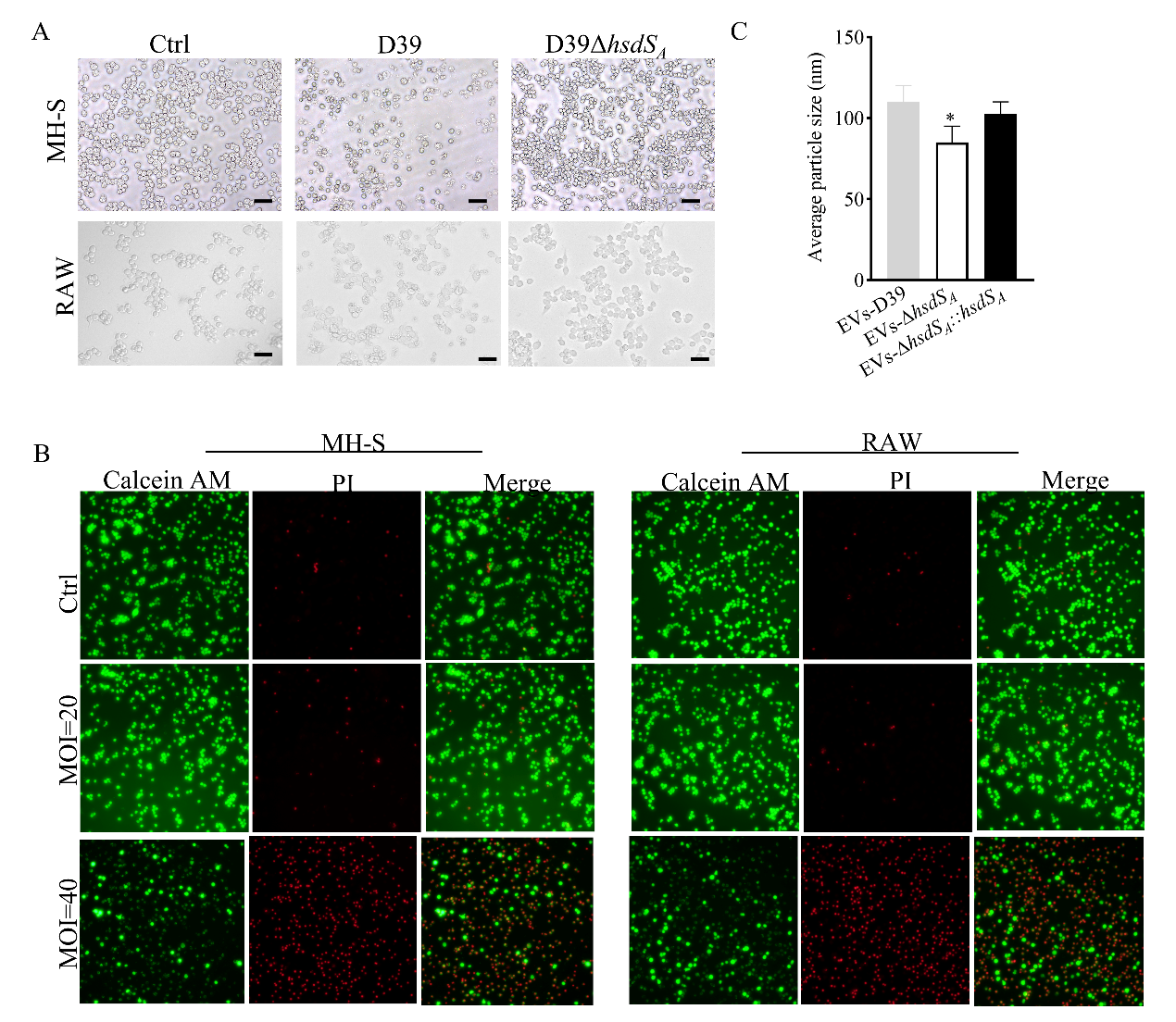
Figure S2 (A) The morphology of macrophages was infected with D39 and D39Δ*hsdS_A_* at a MOI=40 for 3 h. (B) Live cell/dead cell staining of macrophages under D39 infection at MOI of 20 or 40 (×100). (C) Nanobrook ZetaPALS potential analyzer for detecting the particle size. Statistical analysis was analyzed by one-way ANOVA followed by Dunnett’s Multiple Comparison. ^*^*P*<0.05, ^**^*P*<0.01 vs D39.


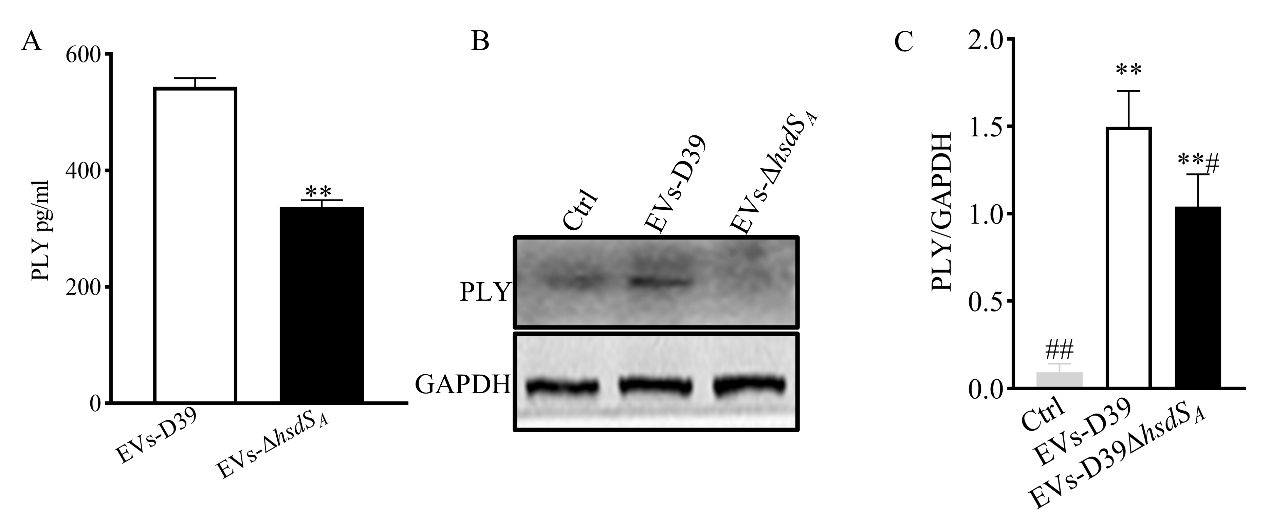

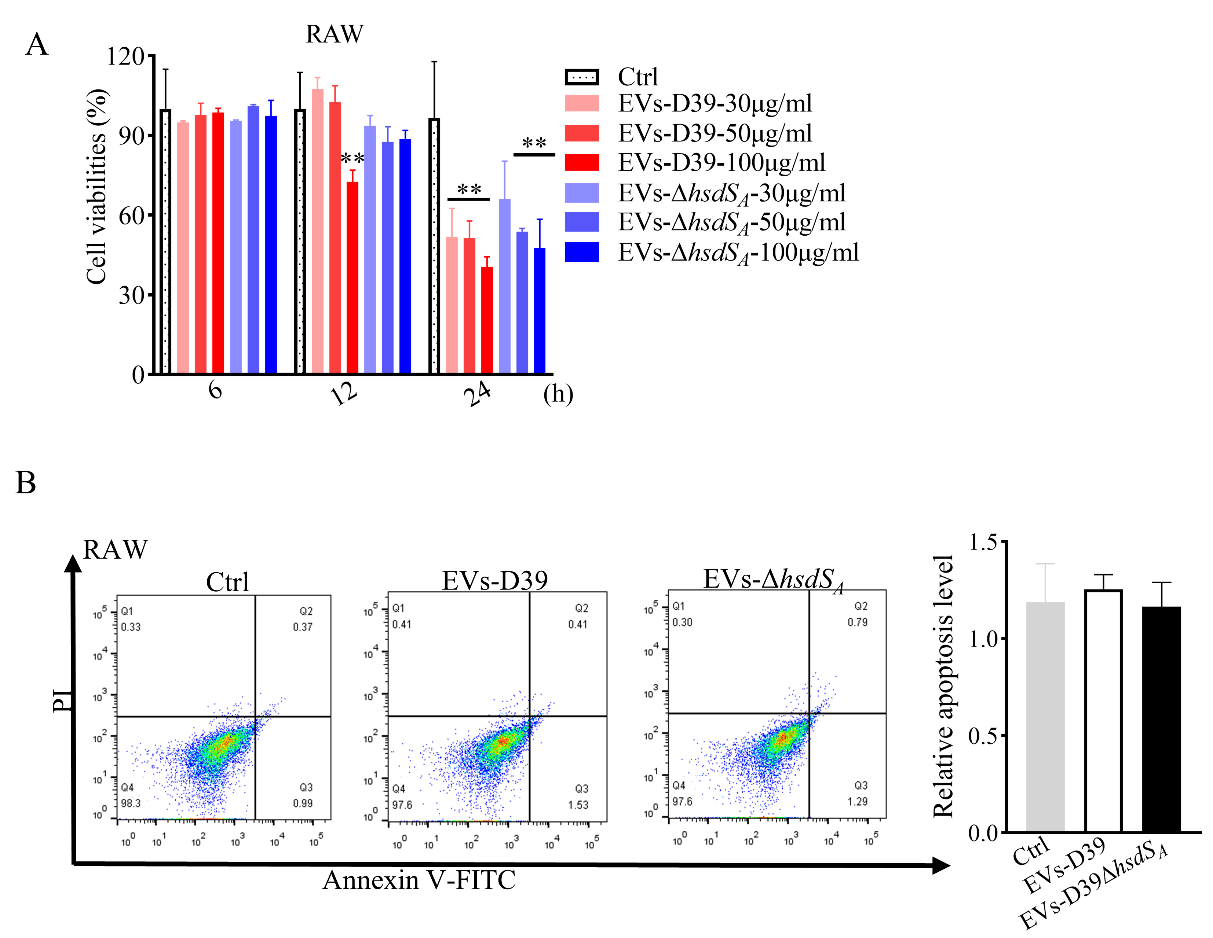
Figure S3 Cytotoxicity assays of EV-associated PLY in RAW264.7. (A) MTT assay for macrophage cell viability in the presence of different concentrations of EVs for 6, 12 and 24 h of coincubation. (B) Representative flow cytometry analysis of macrophage apoptosis with 100 μg EV stimulation for 24 h. Statistics were analyzed by one-way analysis of ANOVA, ^*^*P*<0.05, ^**^*P*<0.01 vs Ctrl.

Figure S4 ELISA (A) and western blot assays for PLY contents in macrophages derived from the internalization of EVs (100 μg) for 6 h (B-C). Data was analyzed by an unpaired Student *t* test (^*^*P*<0.05, ^**^*P*<0.01 vs EVs-D39) or one-way ANOVA followed by Dunnett’s Multiple Comparison (^*^*P*<0.05, ^**^*P*<0.01 vs Ctrl; ^#^*P*<0.05, ^##^*P*<0.01 vs EVs-D39).


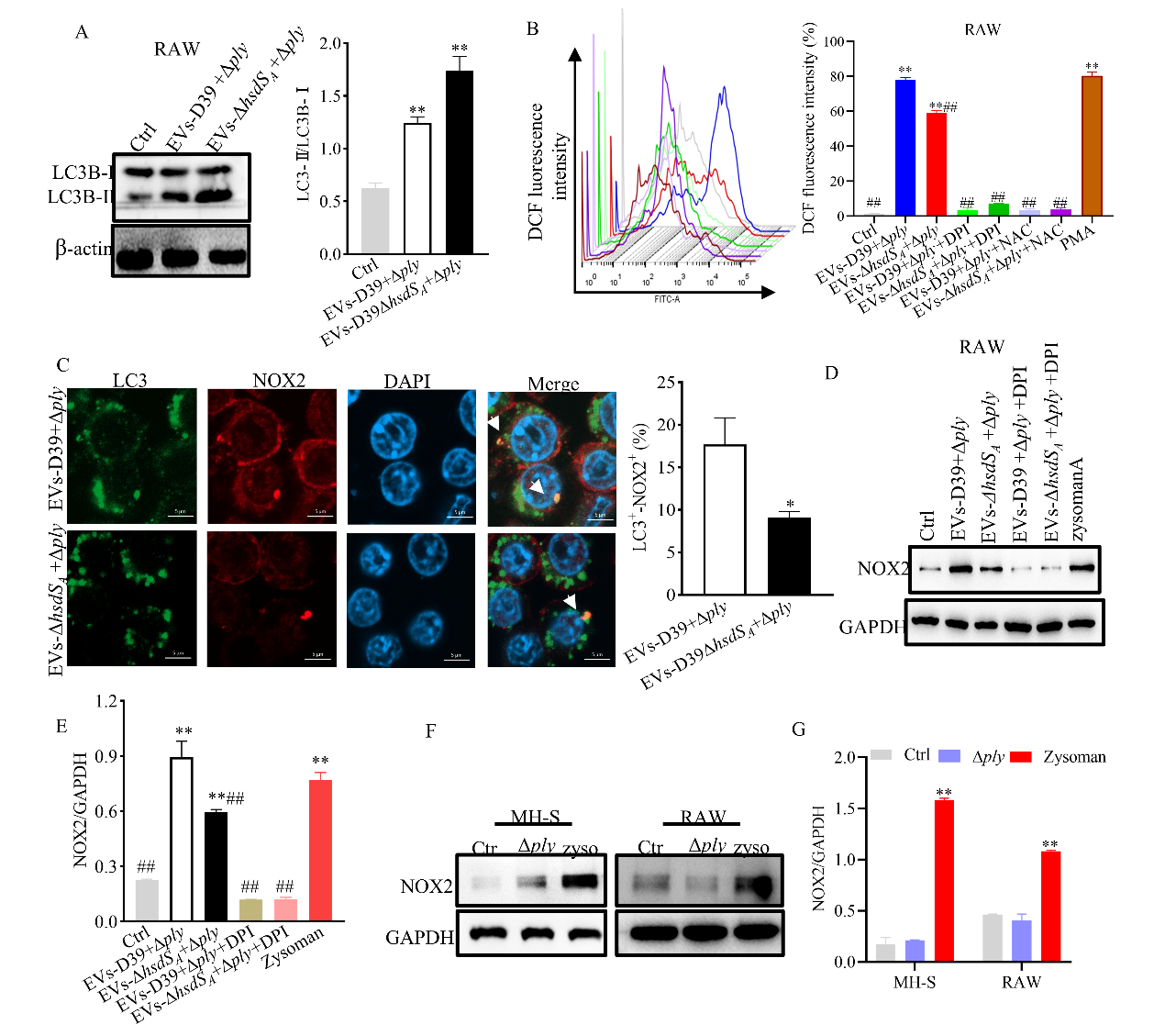
Figure S5 D39 regulated NADPH oxidase-mediated activation of LAP. RAW264.7 was pretreated with EVs for 4 h and infected with D39Δ*ply* for 2 h. (A) Western blot assay for the conversion of LC3-I to LC3-II. Statistical analysis was analyzed by one-way ANOVA followed by Dunnett’s Multiple Comparison. ^*^*P*<0.05, ^**^*P*<0.01 vs Ctrl. (B) Representative flow cytometry analysis of ROS release. DCF fluorescence intensity was quantified. Statistical analysis was analyzed by one-way ANOVA followed by Dunnett’s Multiple Comparison. ^*^*P*<0.05, ^**^*P*<0.01 vs Ctrl; ^#^*P*<0.05, ^##^*P*<0.01 vs EVs-D39+ D39Δ*ply*. (C) Colocalization of LC3B-Ⅰ/Ⅱ and NOX2 were performed by fluorescence microscopy, and quantification was performed by Image J analysis; White arrows represent LC3 colocalization points with NOX2. Statistical analysis was analyzed by an unpaired Student *t* test. ^*^*P*<0.05, ^**^*P*<0.01 vs EVs-D39+ D39Δ*ply*. (D-E) Western blot assay for the expression levels of NOX2. Statistical analysis was analyzed by one-way ANOVA followed by Dunnett’s Multiple Comparison. ^*^*P*<0.05, ^**^*P*<0.01 vs Ctrl; ^#^*P*<0.05, ^##^*P*<0.01 vs EVs-D39+ D39Δ*ply*. (F-G) Western blot assay for the expression levels of NOX2 in MH-S and RAW264.7 infected with D39Δ*ply* for 2 h. Statistical analysis was analyzed by one-way ANOVA followed by Dunnett’s *
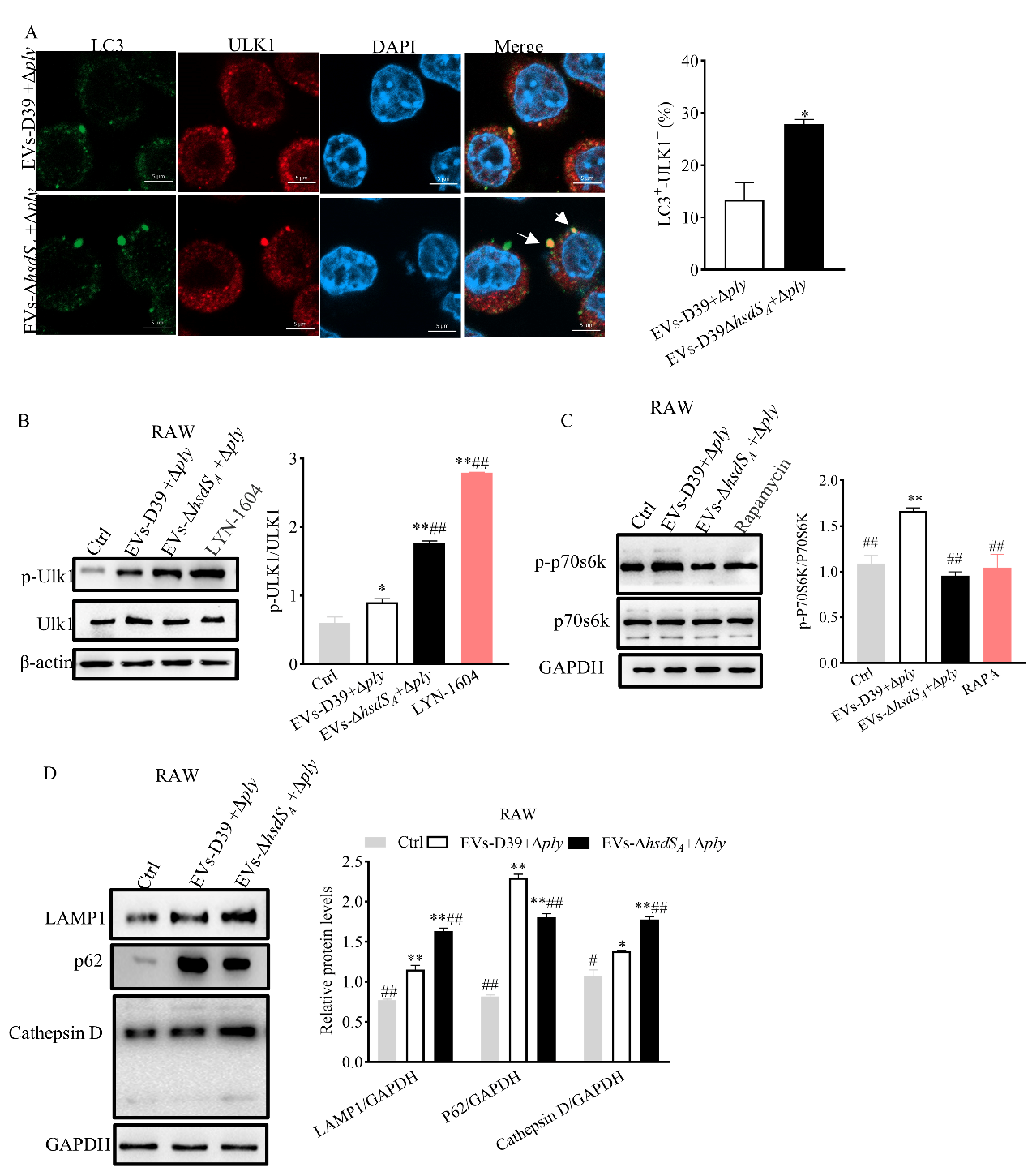
*Multiple Comparison. ^*^*P*<0.05, ^**^*P*<0.01 vs Ctrl.

Figure S6 Effect of EVs-associated PLY on Xenophagy formation. (A) Colocalization of LC3B-Ⅰ/Ⅱ and ULK1 were performed by fluorescence microscopy, and quantification was performed by Image J analysis; White arrows represent LC3 colocalization points with ULK1. Statistical analysis was analyzed by an unpaired Student *t* test. ^*^*P*<0.05, ^**^*P*<0.01 vs EVs-D39+ D39Δ*ply*. (B-D) expression of p-ULK1, p-p70S6K, LAMP1, p62, and cathepsin D were analyzed by western blot. Statistical analysis was analyzed by an unpaired Student *t* test and performed using prism 8.0, ^*^*P*<0.05, ^**^*P*<0.01, EVs-D39+Δ*ply* vs EVs-D39Δ*hsdS_A_*+Δ*ply*. All statistical analysis was analyzed by one-way ANOVA followed by Dunnett’s Multiple Comparison.
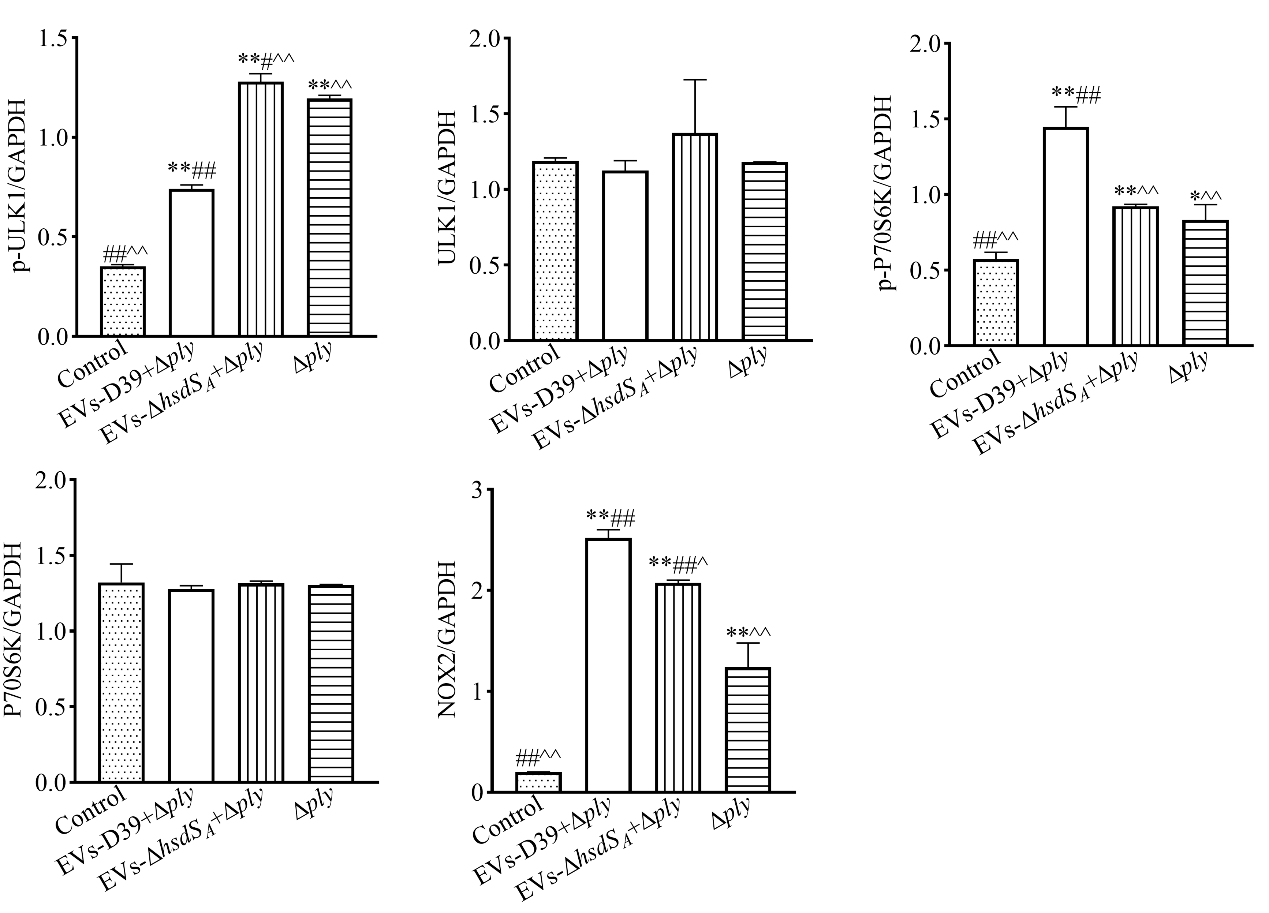
^*^*P*<0.05, ^**^*P*<0.01 vs Ctrl; ^#^*P*<0.05, ^##^*P*<0.01 vs EVs-D39+ D39Δ*ply*.

Figure S7 The expression of LC3B, NOX2, ULK1, p-ULK1, P70S6K and p-P70S6K in mouse alveolar macrophages. All statistical analysis was analyzed by one-way ANOVA followed by Dunnett’s Multiple Comparison. ^*^*P*<0.05, ^**^*P*<0.01 vs Ctrl; ^#^*P*<0.05, ^##^*P*<0.01 vs Δ*ply*; ^^^*P*<0.05, ^^^^*P*<0.01 vs EVs-D39+D39Δ*ply*.
